# Investigating the association of *CD36* gene polymorphisms (rs1761667 and rs1527483) with T2DM and dyslipidemia: Statistical analysis, machine learning based prediction, and meta-analysis

**DOI:** 10.1371/journal.pone.0257857

**Published:** 2021-10-14

**Authors:** Ma’mon M. Hatmal, Walhan Alshaer, Ismail S. Mahmoud, Mohammad A. I. Al-Hatamleh, Hamzeh J. Al-Ameer, Omar Abuyaman, Malek Zihlif, Rohimah Mohamud, Mais Darras, Mohammad Al Shhab, Rand Abu-Raideh, Hilweh Ismail, Ali Al-Hamadi, Ali Abdelhay

**Affiliations:** 1 Department of Medical Laboratory Sciences, Faculty of Applied Medical Sciences, The Hashemite University, Zarqa, Jordan; 2 Cell Therapy Centre, The University of Jordan, Amman, Jordan; 3 Department of Immunology, School of Medical Sciences, Universiti Sains Malaysia, Kubang Kerian, Kelantan, Malaysia; 4 Department of Biology and Biotechnology, American University of Madaba, Madaba, Jordan; 5 Department of Pharmacology, Faculty of Medicine, The University of Jordan, Amman, Jordan; Indiana University Purdue University at Indianapolis, UNITED STATES

## Abstract

CD36 (cluster of differentiation 36) is a membrane protein involved in lipid metabolism and has been linked to pathological conditions associated with metabolic disorders, such as diabetes and dyslipidemia. A case-control study was conducted and included 177 patients with type-2 diabetes mellitus (T2DM) and 173 control subjects to study the involvement of *CD36* gene rs1761667 (G>A) and rs1527483 (C>T) polymorphisms in the pathogenesis of T2DM and dyslipidemia among Jordanian population. Lipid profile, blood sugar, gender and age were measured and recorded. Also, genotyping analysis for both polymorphisms was performed. Following statistical analysis, 10 different neural networks and machine learning (ML) tools were used to predict subjects with diabetes or dyslipidemia. Towards further understanding of the role of CD36 protein and gene in T2DM and dyslipidemia, a protein-protein interaction network and meta-analysis were carried out. For both polymorphisms, the genotypic frequencies were not significantly different between the two groups (*p* > 0.05). On the other hand, some ML tools like multilayer perceptron gave high prediction accuracy (≥ 0.75) and Cohen’s kappa (κ) (≥ 0.5). Interestingly, in K-star tool, the accuracy and Cohen’s κ values were enhanced by including the genotyping results as inputs (0.73 and 0.46, respectively, compared to 0.67 and 0.34 without including them). This study confirmed, for the first time, that there is no association between *CD36* polymorphisms and T2DM or dyslipidemia among Jordanian population. Prediction of T2DM and dyslipidemia, using these extensive ML tools and based on such input data, is a promising approach for developing diagnostic and prognostic prediction models for a wide spectrum of diseases, especially based on large medical databases.

## Introduction

Diabetes mellitus (DM) is a metabolic disorder characterized by high levels of blood glucose due to defective insulin production, insulin action, or both [1]. If remained uncontrolled, diabetes could lead to serious health complications that affect various systems of human body including blood vessel and nervous system damage, vision complications, cardiovascular disease, and infection [1]. Diabetes affects millions of people worldwide. In 2014, it was reported that nearly 380 million people worldwide had the disease [2]; this number is constantly increasing, and is expected to grow tremendously in the future. Type-2 DM (T2DM) is the prevalent form of diabetes, which accounts approximately 90% of all diagnosed cases of diabetes in adults [3]. T2DM is mainly manifested by low insulin production by pancreatic cells and/or the produced insulin does not function effectively [4]. Many genetic factors and polymorphisms have been investigated in patients with T2DM; we have previously investigated that the *vitamin D receptor* (*VDR*) gene *FokI* polymorphism, the DNA-binding domain of *regulatory factor X6* (*RFX6*) gene, as well as the *epoxide hydrolase* (*EPHX2*) gene rs4149243, rs2234914 and rs751142 variants [5–7].

**Fig 1:**
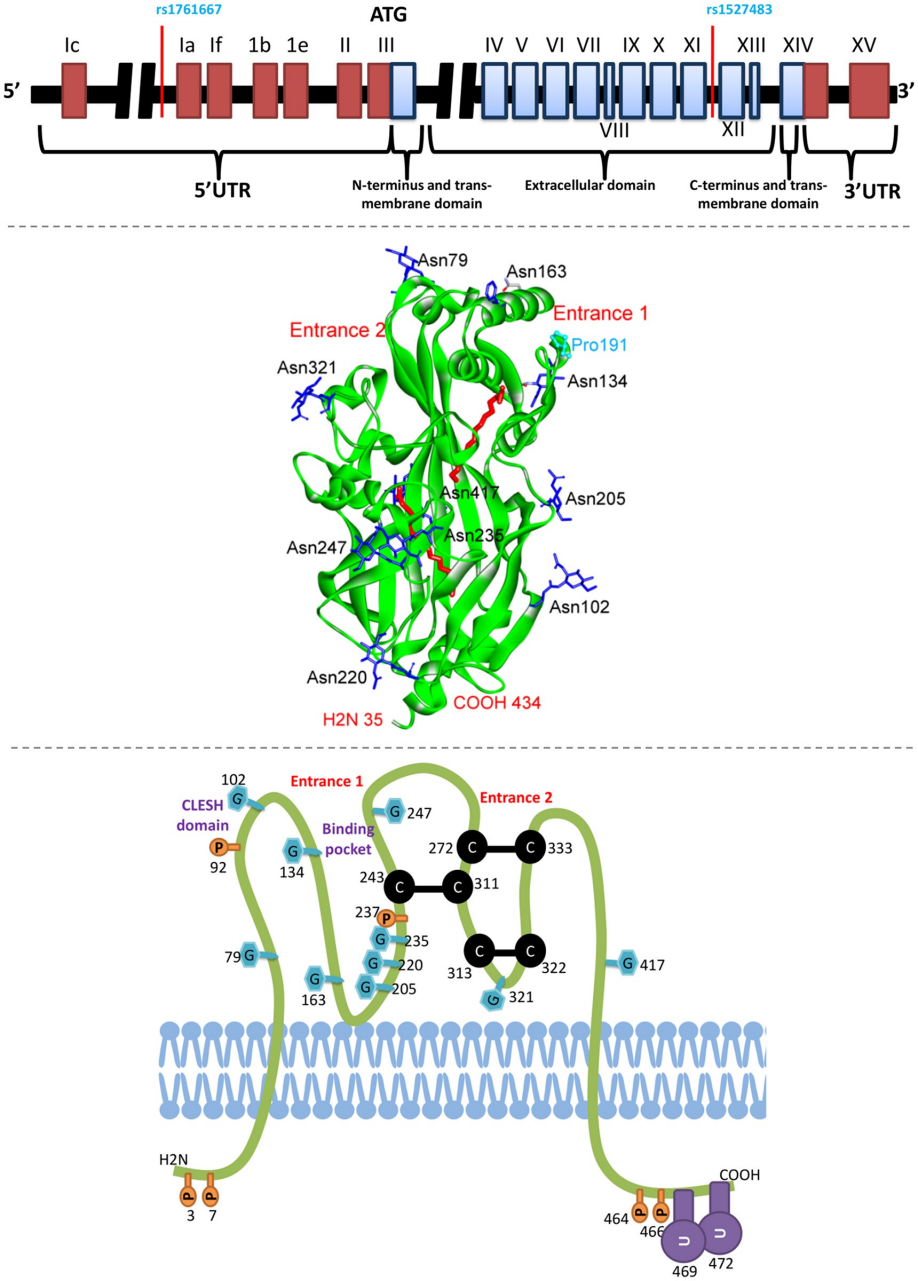
Representation of *CD36* gene and protein structure. The upper illustration shows the gene composition of CD36; the encoding boxes are blue colored, the locations of rs1761667 and rs1527483 polymorphisms are indicated [12]. Based on a structure file obtained from “protein data bank (PDB)” (PDB ID: 5LGD), the middle illustration shows the three dimensional structure of CD36 protein extracellular domain with assigning the main two entrances for fatty acids (indicated in red color). The blue residues are NAG gylocsylated asparagines residues. Pro191 is a mutation at the frontage of entrance 1 which affects binding with fatty acids [13]. The lower illustration shows the 2-D representation of CD36 protein and the location of different modifications and disulfide bridges; G stands for glycosylation, C-C stands for disulfide bond, U for ubiquitination, and P for phosphorylation [12].

CD36 is a membrane glycoprotein receptor that is expressed on a variety of cells and tissues, including platelets, macrophages, adipocytes, hepatocytes, myocytes, and some specialized epithelia of the breast, kidney and gut [8]. The genetic composition, 2D and 3D protein structures of CD36 are shown in Fig 1. CD36 is a multifunctional signaling receptor with several known ligands, including thrombospondin-1, long chain fatty acids, oxidized low-density and high-density lipoproteins (LDL and HDL) [9]. In macrophages, CD36 acts as a scavenger receptor that recognizes specific oxidized phospholipids and LDL, as well as participates in internalization of apoptotic cells and certain bacterial and fungal pathogens, contributing to inflammatory responses and atherothrombotic diseases [8]. Also, CD36 functions on adipocytes, enterocytes, hepatocytes and muscles as a facilitator of long-chain fatty acid transport participating in intestinal fat absorption, muscle lipid utilization, and adipose energy storage [10, 11].

It has been shown that CD36 is involved in lipid metabolism and homoeostasis and has been linked to pathological conditions associated with metabolic disorders, such as obesity, insulin resistance, diabetes, dyslipidemia and atherosclerosis [9, 14–17]. The mechanistic role of CD36 in metabolic diseases is seemed to be complex and yet to be resolved. However, the contribution of CD36 in mediating cellular lipid transport and intracellular accumulation of lipid is expected to cause lipotoxicity and, hence, insulin dysregulation and resistance [18].

CD36 protein is encoded by a gene which is located on chromosome 7q11.2 and has 15 exons [19]. It has been reported that genetic mutations in *CD36* gene could be associated with the pathogenesis of T2DM [20–23]. The rs1761667 (G>A) and rs1527483 (C>T) polymorphisms are two main single nucleotide polymorphisms (SNPs) in the *CD36* gene that have been previously studied in T2DM [24]. The aim of the current study is to assess potential association between the rs1761667 and rs1527483 polymorphisms with T2DM and dyslipidemia in Jordanian population. Although dyslipidemia was shown by previous studies to be associated with T2DM, it is not necessary that every patient with T2DM must have dyslipidemia [25, 26].

On the other hand, recent years have witnessed an unprecedented development in the use of machine learning (ML) in various biotechnology, biomedicine, medical imaging and healthcare applications [27–30]. Supervised ML tools can be utilized to build predictive models involve the implementation of statistical means for learning and predicting disease status, either by including or excluding the polymorphisms genotypes [5, 31–33]. The following are popular ML algorithms that were evaluated in the current research to predict T2DM and dyslipidemia based on the clinical parameters, demographic and polymorphism data: random forest (RF) [34–36]; naïve Bayesian (NB) [37–40]; eXtreme Gradient Boosting (XGBoost) [41–43]; k-nearest neighbors (kNN) [44–46], support vector machine (SVM) [47, 48]; probabilistic neural networks (PNN) [49–53]; multilayer perceptron (MLP) [54, 55]; adaptive boosting (AdaBoost) [56, 57]; gradient boost [58, 59]; and K-star (K*) [60, 61]. It was reported that the odds ratio for each T2DM risk allele varies between 1.02 and 1.35. To produce improved prediction results for complicated polygenic traits, recent polygenic risk score models integrate expanded SNP selection [62, 63]. The goal of this research is to see how a small number of polymorphisms can improve machine learning prediction based on clinical and demographic data. However, ML needs to be validated vis-à-vis statistical accuracy (i.e., predictability). Moreover, a protein-protein interaction network was used towards further understanding of CD36 interactions. Also, to compare our results with the previous findings, the first meta-analysis for the association of these two polymorphisms with diabetes was performed.

## Results

### Demographic and clinical data

**Fig 2:**
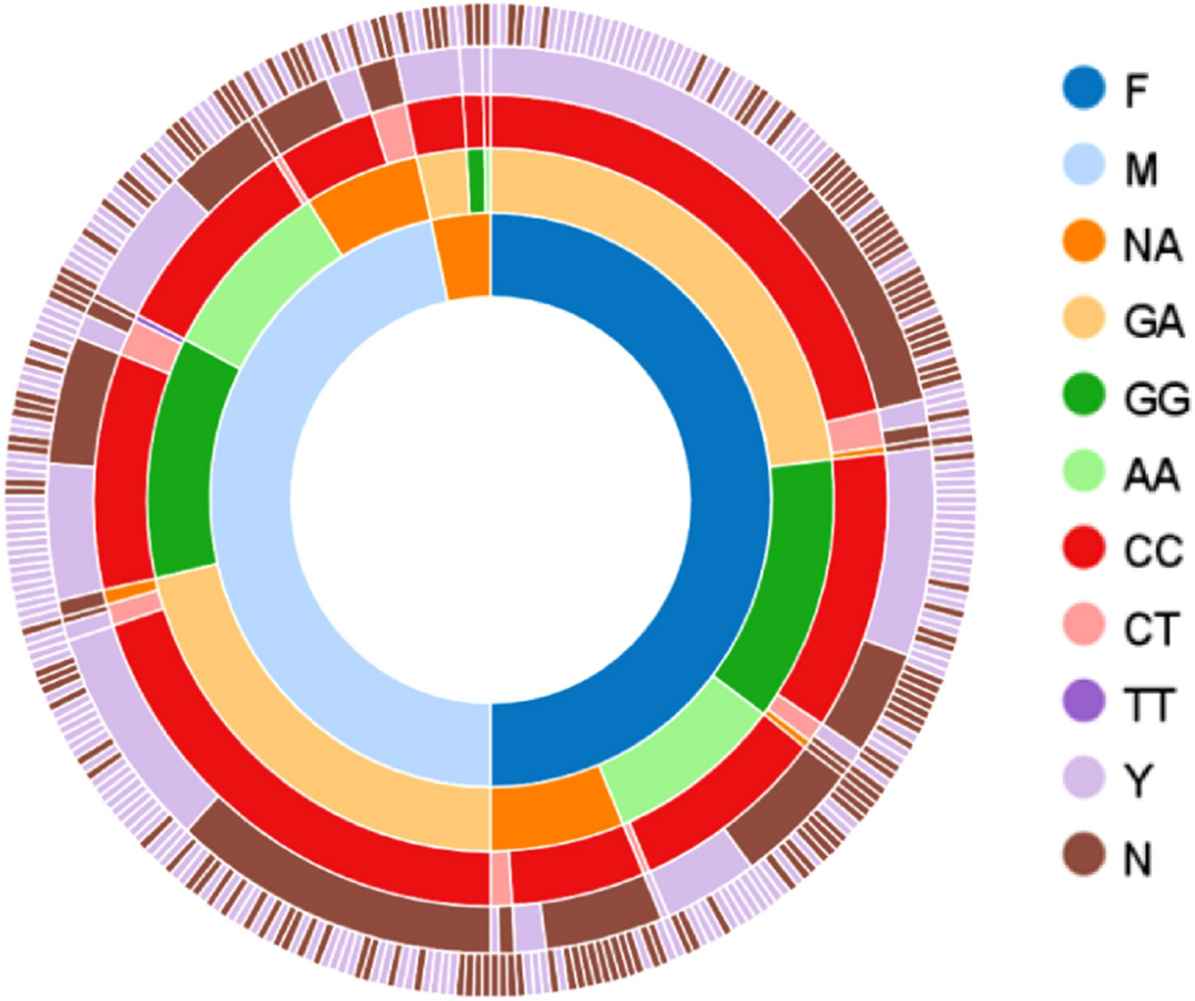
Sunburst figure shows the distribution of all samples (*n* = 350) among different categories. The first inner circle indicates the gender (175 females (F), 163 males (M), and 12 not available (NA)), while the second and third circles show different genotypes of rs1761667 (*n* = 310) and rs1527483 (*n* = 347), respectively. The fourth and fifth ones represent diabetes (yes (Y) = 177, and no (N) = 173) and dyslipidemia (Y = 201, and N = 149) status, respectively.

The average (standard deviation (SD)) of age for T2DM patients (*n* = 177) and control group (*n* = 173) were 50.8 (13.9) years and 57.4 (11.6) years, respectively. Based on t-test, there was no significant difference between the two groups. The distribution of the data among categorical classes is indicated briefly in Fig 2. Baseline data of the study subjects is available in S1 Table. The differences between T2DM and control groups based on age, gender, FBS, and lipid parameters are shown in Table 1, and none of them has shown significant differences between the two groups.

**Table 1:** Demographic and clinical characteristic of 350 Jordanian subjects participated in the study.

Sample characteristics	Controls (*n* = 173) (Avg. ± STD)	T2DM Patients (*n* = 177) (Avg. ± STD)	*p*-value
Age (years)	50.81 ± 13.94	57.33 ± 11.56	> 0.05*
Gender	F (81), M (92)	F (94), M (71)	> 0.05**
TC (mg/dl)	198.35 ± 37.72	178.71 ± 46.70	> 0.05*
TG (mg/dl)	142.59 ± 76.54	212.99 ± 103.20	> 0.05*
HDL (mg/dl)	53.29 ± 31.21	43.76 ± 22.98	> 0.05*
LDL (mg/dl)	120.13 ± 32.25	93.72 ± 48.22	> 0.05*
FBS (mg/dl)	99.29 ± 11.43	199.67 ± 88.38	> 0.05*

HDL, high-density lipoprotein; LDL, low-density lipoprotein; TC, total cholesterol; TG, triglycerides; FBS, fasting blood sugar; F, female; M, male.

* and ** refer to the *p*-value resulted from t-test and chi-square test, respectively (*p*-value is significant if < 0.05).

Some people have missing data as indicated in S1 Table.

Table 2 shows the distribution of samples used in the present study based on their *CD36* polymorphisms. The genotypic and allelic frequencies, as well as the exact tests for Hardy-Weinberg equilibrium are shown in S2 Table. The D value for Linkage disequilibrium analysis between the two SNPs is very low (i.e., −0.01), which indicates that the gamete is not more frequent than expected. Table 3 shows odd ratio and its related *p*-values for different genotypes for rs1761667 in T2DM based on different genetic models, while Table 4 shows them for rs1527483. None of the genotypes for all genetic models was significantly different between T2DM and control groups. The stratified distribution among females and males, and their corresponding odd ratio and *p*-value, are shown in S3 and S4 Tables for rs1761667 and rs1527483, respectively.

**Table 2:** Frequency distribution of samples used in this study on Jordanian population with *CD36* polymorphisms (rs1761667 and rs1527483).

Sample type	rs1761667 (G>A) n (%)	rs1527483 (C>T) n (%)
Control	139 (44.8)	169 (48.7)
T2DM	171 (55.2)	178 (51.3)
Total	310 (100)	347 (100)

**Table 3:** Polymorphism rs1761667 association with response diabetes based on "SNPStats" analysis tool.

Model	Genotype	Control	T2DM	OR (95% CI)	*p-*value
Codominant	GG	28 (27.2%)	36 (27.7%)	1	0.52
	GA	54 (52.4%)	69 (53.1%)	1.46 (0.49–4.41)	
	AA	21 (20.4%)	25 (19.2%)	2.24 (0.56–9.01)	
Dominant	GG	28 (27.2%)	36 (27.7%)	1	0.36
	GA-AA	75 (72.8%)	94 (72.3%)	1.63 (0.56–4.71)	
Recessive	GG-GA	82 (79.6%)	105 (80.8%)	1	0.36
	AA	21 (20.4%)	25 (19.2%)	1.72 (0.55–5.40)	
Overdominant	GG-AA	49 (47.6%)	61 (46.9%)	1	0.94
	GA	54 (52.4%)	69 (53.1%)	1.03 (0.42–2.55)	
Log-additive	---	---	---	1.50 (0.75–2.99)	0.25

*p*-value obtained based on odd-ration (OR) test.

**Table 4:** Polymorphism rs1527483 association with response diabetes based on "SNPStats" analysis tool.

Model	Genotype	Control	T2DM	OR (95% CI)	*p*-value
Codominant	CC	114 (92.7%)	123 (91.8%)	1.00	0.56
	CT	8 (6.5%)	11 (8.2%)	2.36 (0.45–12.41)	
	TT	1 (0.8%)	0 (0%)	0.00 (0.00-NA)	
Dominant	CC	114 (92.7%)	123 (91.8%)	1.00	0.37
	CT-TT	9 (7.3%)	11 (8.2%)	2.14 (0.42–10.88)	
Recessive	CC-CT	122 (99.2%)	134 (100%)	1.00	0.66
	TT	1 (0.8%)	0 (0%)	0.00 (0.00-NA)	
Overdominant	CC-TT	115 (93.5%)	123 (91.8%)	1.00	0.32
	CT	8 (6.5%)	11 (8.2%)	2.38 (0.45–12.54)	
Log-additive	---	---	---	1.76 (0.42–7.35)	0.46

*p*-value obtained based on odd-ration (OR) test.

Haplotype frequencies among all people in the study are shown in S5 Table and their gender cross-classification is shown in S6 Table. Based on odd-ration test and its *p*-value, haplotype association with T2DM is shown in Table 5, and their frequencies among females and males are indicated. None of the haplotypes was significantly different between T2DM and control groups.

**Table 5:** Haplotype association with T2DM based on "SNPStats" analysis tool.

rs1761667	rs1527483	Frequency	OR (95% CI)	*p*-value
G	C	0.5068	1.00	---
A	C	0.4524	1.76 (0.86–3.59)	0.12
G	T	0.0366	2.43 (0.50–11.80)	0.27
A	T	0.0042	2.65 (0.00–13287.22)	0.82

*p*-value obtained based on odd-ration (OR) test. Global haplotype association *p*-value: 0.39.

On the other hand, Table 6 shows odd ratio and *p*-values for different genotypes for rs1761667 in dyslipidemia based on different genetic models, while Table 7 shows them for rs1527483. The stratified distribution among females and males are shown in S7 and S8 Tables for rs1761667 and rs1527483, respectively.

**Table 6:** Polymorphism rs1761667 association with response dyslipidemia based on "SNPStats" analysis tool.

Model	Genotype	No-dyslipidemia	Dyslipidemia	OR (95% CI)	*p-*value
Codominant	GG	23 (24.2%)	41 (29.7%)	1.00	0.25
	GA	52 (54.7%)	71 (51.5%)	0.50 (0.20–1.21)	
	AA	20 (21.1%)	26 (18.8%)	0.81 (0.27–2.39)	
Dominant	GG	23 (24.2%)	41 (29.7%)	1.00	0.19
	GA-AA	72 (75.8%)	97 (70.3%)	0.57 (0.25–1.33)	
Recessive	GG-GA	75 (79%)	112 (81.2%)	1.00	0.59
	AA	20 (21.1%)	26 (18.8%)	1.28 (0.52–3.16)	
Overdominant	GG-AA	43 (45.3%)	67 (48.5%)	1.00	0.11
	GA	52 (54.7%)	71 (51.5%)	0.55 (0.26–1.15)	
Log-additive	---	---	---	0.87 (0.51–1.48)	0.6

*p*-value obtained based on odd-ration (OR) test.

**Table 7:** Polymorphism rs1527483 association with response dyslipidemia based on "SNPStats" analysis tool.

Model	Genotype	No-dyslipidemia	dyslipidemia	OR (95% CI)	*p*-value
Codominant	CC	102 (91.1%)	135 (93.1%)	1.00	0.69
	CT	9 (8%)	10 (6.9%)	1.35 (0.35–5.22)	
	TT	1 (0.9%)	0 (0%)	0.00 (0.00-NA)	
Dominant	CC	102 (91.1%)	135 (93.1%)	1.00	0.79
	CT-TT	10 (8.9%)	10 (6.9%)	1.20 (0.32–4.43)	
Recessive	CC-CT	111 (99.1%)	145 (100%)	1.00	0.46
	TT	1 (0.9%)	0 (0%)	0.00 (0.00-NA)	
Overdominant	CC-TT	103 (92%)	135 (93.1%)	1.00	0.66
	CT	9 (8%)	10 (6.9%)	1.36 (0.35–5.28)	
Log-additive	---	---	---	1.06 (0.33–3.44)	0.92

*p*-value obtained based on odd-ration (OR) test.

### Machine learning

#### Principal Component Analysis (PCA)

**Fig 3:**
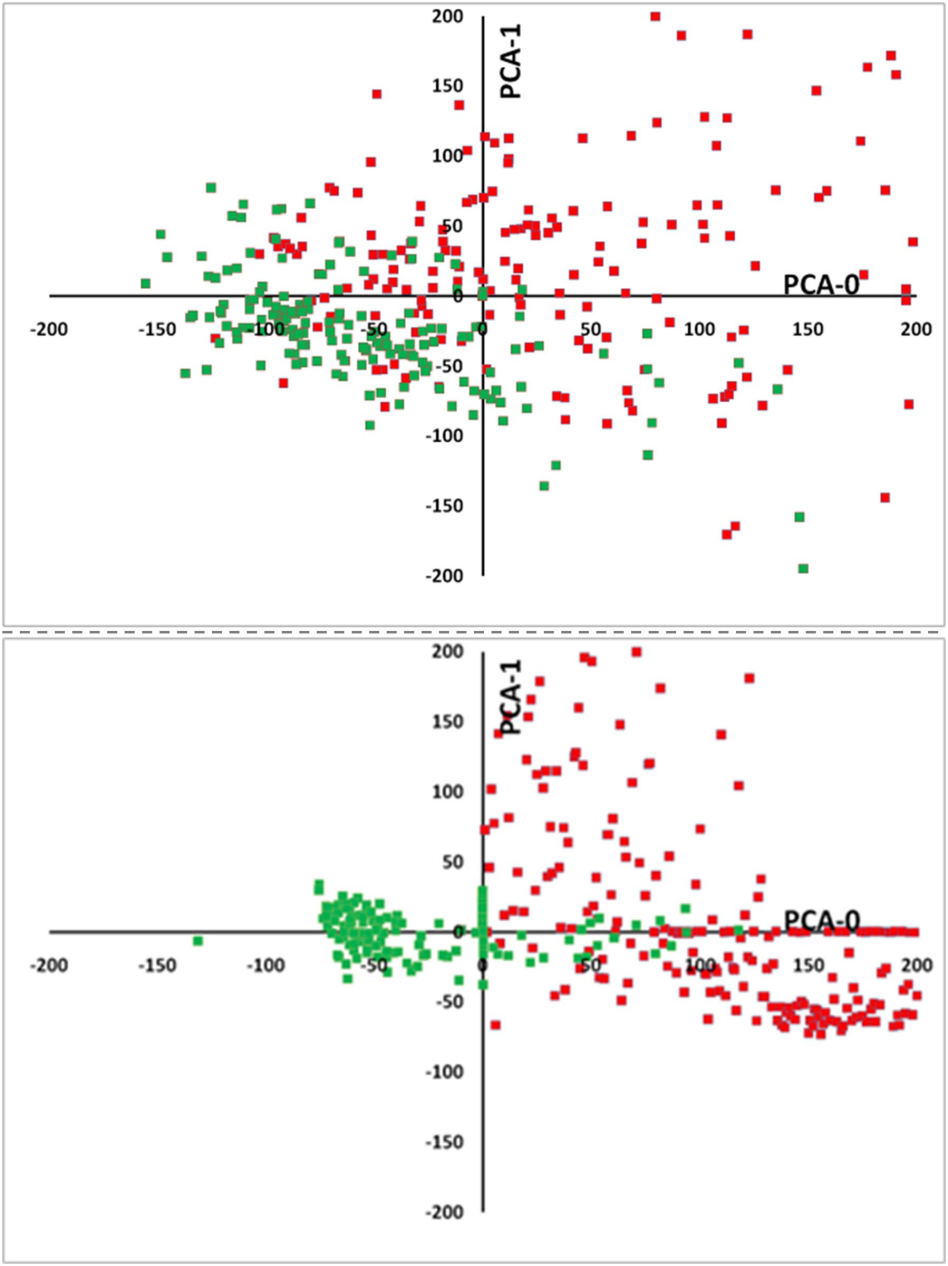
Two-dimensional plots showing two main principal components. In the upper graph, five input features (LDL, HDL, TG, TC and Age) were calculated for subjects with diabetes (red squares) compared to all control subjects (green squares). In the lower graph, two input features (FBS and Age) were calculated for all subjects with dyslipidemia (red squares) compared to all control subjects (green squares). The clinical parameters used to define the diseases (lipid profile for dyslipidemia, and FBS for T2DM) were excluded from the input data as described before.

PCA function performs a principal component analysis (PCA) on the given data. The input data is projected from its original feature space into a space of (possibly) lower dimension with a minimum of information loss. Fig 3 represents the PCA for the normal subjects (status = 0, green color) and subjects with disease (status = 1, red color).

#### Predicting diabetes and dyslipidemia for the testing set using different ML models

**Fig 4:**
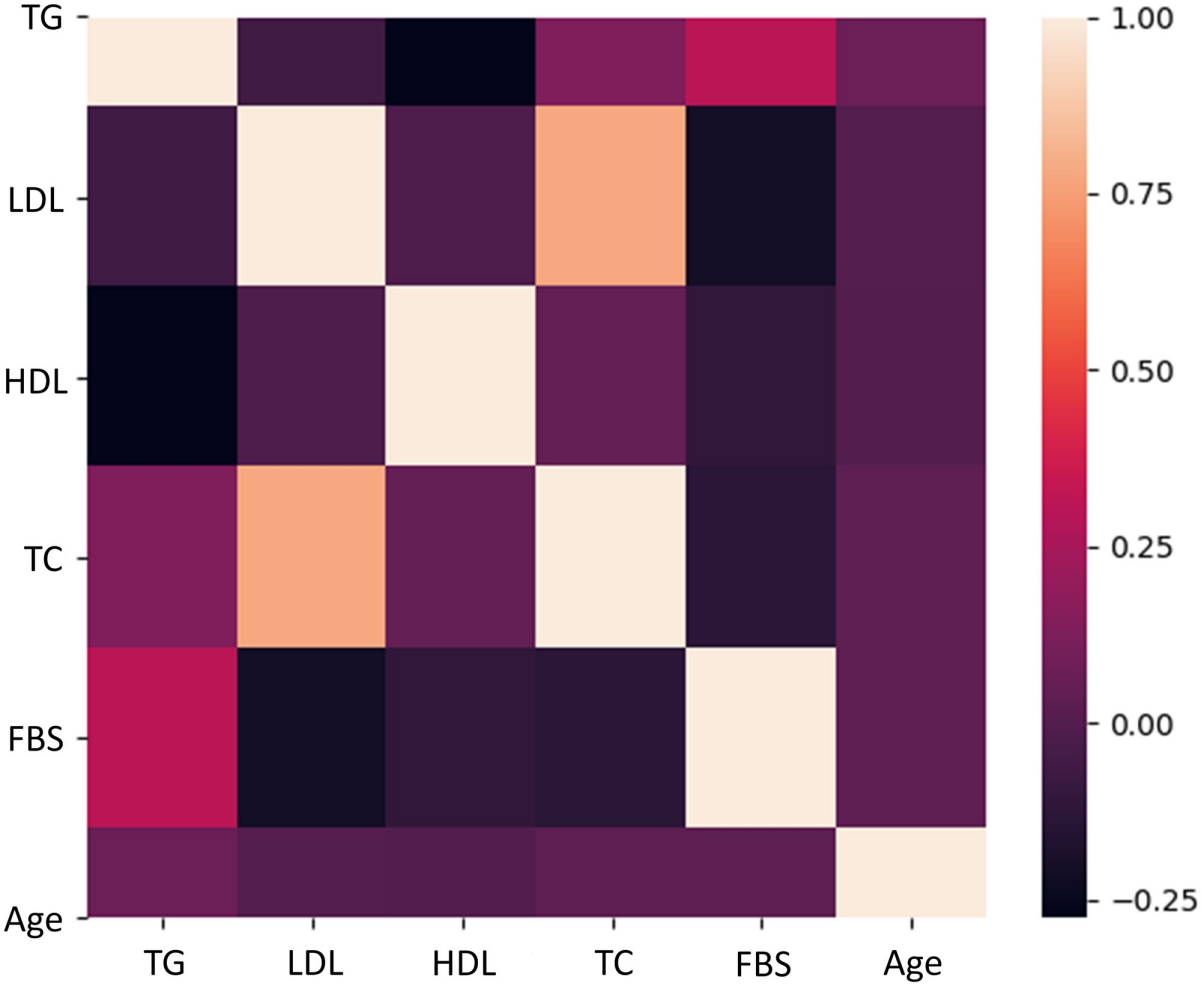
A heat map shows the associations between Fasting Blood Sugar (FBS), age and lipid parameters. Color scale is displayed at the right corner. A positive correlation is indicated by light colors (i.e. yellow), while a negative relationship is indicated by dark colors (i.e., dark purple). Triglyceride (TG) is moderately associated with FBS, needless to say that there is a strong correlation between low-density lipoprotein (LDL) and TC.

A heat map was generated for all parameters (input and output), except for those that have either binomial or discrete values (gender and polymorphisms). TG is moderately associated with BS, and a strong correlation between LDL and TC is clearly presented (Fig 4). Pearson’s correlation (r, as shown on Y axis of Fig 4) was used as measure for correlations; r > 0.7 is considered strong correlation, between 0.4 and 0.7 moderately correlated, and < 0.4 weakly correlated [64].

For prediction using different ML tools, a confusion matrix was built for the testing set for each group of the selected inputs (Table 8). Number of people with the disease who were randomly selected in the testing set is "a + c", and number of people with no disease randomly selected in the testing set is "b + d".

**Table 8:** General shape of the confusion matrix.

	Disease/condition	No condition
Predicted condition	a	b
Predicted no condition	c	d
	a + c	b + d

"a" represents the true predicted patients with the disease, "b" represents the false predicted people with the disease, "c" represents the false predicted people with no disease, and "d" represents the true predicted people with no disease. "a + c" represents the total number of people with the disease, while "b + d" represents the total number of people with no disease.

Accuracy was calculated according to the following formula (Eq 1):

Accuracy=(TP+TN)/(TP+TN+FP+FN)=(a+d)/(a+b+c+d) (1)

where TP is true positive; TN is true negative; FP is false positive; and FN is false negative. True positive rate (TPR or sensitivity) was calculated by the formula (Eq 2):

$$TPR=\frac{TP}{TP+FN}=\frac{a}{a+c}$$ (2)

while true negative rate (TNR or specificity) was calculated by the formula (Eq 3):

$$TNR=\frac{TN}{TN+FP}=\frac{d}{b+d}$$ (3)

Table 9 shows the accuracy, cohen’s κ, TPR and TNR values to predict T2DM of different ML tools for both 5-fold cross validation and 20% testing sets based on all input data: lipid profile (TG, TC, LDL, and HDL), dyslipidemia status, age, gender, rs1761667 genotype, and rs1527483 genotype, while Table 10 shows the prediction results based on all input data used in Table 9, excluding the polymorphisms genotypes. MLP was also used to predict dyslipidemia for both 5-fold cross validation and 20% testing sets based on all input data and data excluding polymorphisms (Table 11).

**Table 9:** Accuracy, cohen’s κ, TPR and TNR values based for the all ML tools and using either 5-fold cross validation or 20% testing set for prediction people with T2DM from people without T2DM.

ML tool	Type of Data	Measure			
		Accuracy	Cohen’s	TPR	TNR
Logistic	5-fold cross validation	0.75	0.50	0.76	0.74
	20% Testing	0.97	0.94	1.00	0.95
RF	5-fold cross validation	0.76	0.51	0.79	0.73
	20% Testing	0.66	0.34	0.97	0.38
XGBoost	5-fold cross validation	0.73	0.64	0.72	0.73
	20% Testing	0.66	0.33	0.94	0.41
PNN	5-fold cross validation	0.62	0.24	0.60	0.64
	20% Testing	0.86	0.71	0.88	0.84
C-LibSVM	5-fold cross validation	0.65	0.31	0.71	0.60
	20% Testing	0.47	0.00	1.00	0.00
nu-LibSVM	5-fold cross validation	0.66	0.32	0.68	0.64
	20% Testing	0.53	0.00	1.00	0.00
AdaBoost	5-fold cross validation	0.74	0.49	0.82	0.61
	20% Testing	0.60	0.23	0.97	0.27
Gradient-boost	5-fold cross validation	0.73	0.46	0.71	0.75
	20% Testing	0.59	0.18	0.64	0.54
KNN*	5-fold cross validation	0.62	0.24	0.54	0.70
	20% Testing	0.47	0.00	1.00	0.00
K-star	5-fold cross validation	0.73	0.46	0.68	0.78
	20% Testing	0.53	0.07	0.67	0.41

* Features with non-numeric values are ignored.

**Table 10:** Accuracy, cohen’s κ, TPR, and TNR values based for the all ML tools and using either 5-fold cross validation or 20% testing set for prediction people with T2DM from people without T2DM.

ML tool	Type of Data	Measure			
		Accuracy	Cohen’s	TPR	TNR
Logistic (MLP)	5-fold cross validation	0.77	0.54	0.75	0.79
	20% Testing	0.99	0.97	1.00	0.97
Random Forest	5-fold cross validation	0.75	0.50	0.75	0.76
	20% Testing	0.64	0.31	0.94	0.38
XGBoost	5-fold cross validation	0.74	0.49	0.77	0.71
	20% Testing	0.69	0.39	0.97	0.43
PNN	5-fold cross validation	0.62	0.24	0.64	0.60
	20% Testing	0.86	0.71	0.88	0.84
C-LibSVM	5-fold cross validation	0.63	0.29	0.67	0.60
	20% Testing	0.47	0.00	1.00	0.00
nu-LibSVM	5-fold cross validation	0.65	0.30	0.63	0.66
	20% Testing	0.53	0.00	0.00	1.00
AdaBoost	5-fold cross validation	0.71	0.42	0.72	0.69
	20% Testing	0.60	0.23	0.97	0.27
Gradient-Boost	5-fold cross validation	0.72	0.45	0.72	0.72
	20% Testing	0.57	0.16	0.79	0.38
KNN*	5-fold cross validation	0.64	0.28	0.60	0.68
	20% Testing	0.47	0.00	1	0.00
K-star	5-fold cross validation	0.67	0.34	0.66	0.68
	20% Testing	0.49	0.00	0.64	0.35

*Features with non-numeric values are ignored.

**Table 11:** Accuracy, cohen’s, TPR, and TNR based on MLP and using either 5-fold cross validation or 20% testing set for prediction people with T2DM from people without dyslipidemia.

Logistic	Type of Data	Measure			
		Accuracy	Cohen’s	TPR	TNR
All input data	5-fold cross validation	0.67	0.35	0.76	0.59
	20% Testing	0.60	0.05	0.98	0.07
Data excluding rs1761667 and rs1527483 genotypes	5-fold cross validation	0.69	0.36	0.71	0.66
	20% Testing	0.59	0.00	1.00	0.00

### Meta-analysis

**Fig 5:**
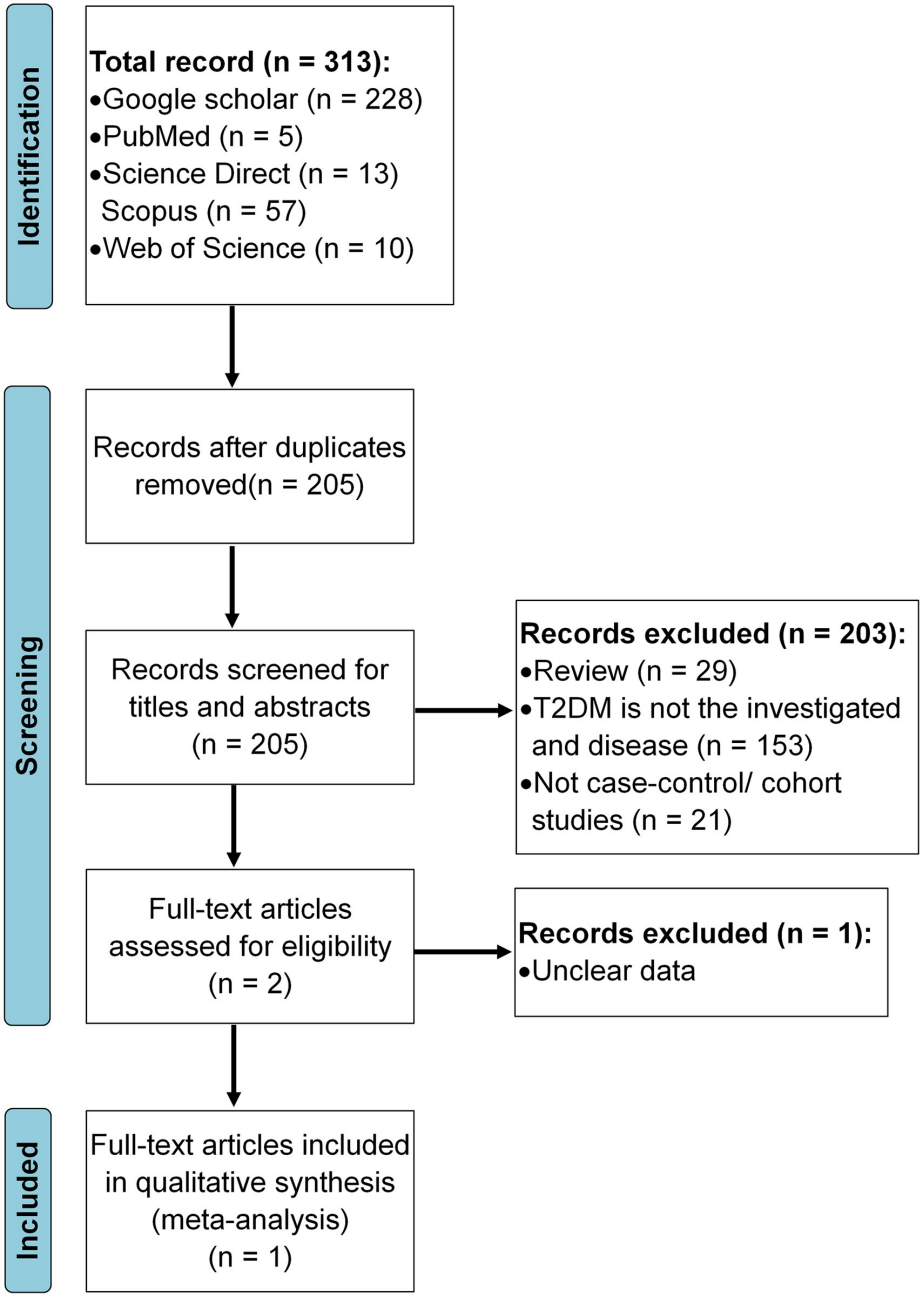
Flowchart of article identification and selection process. This figure prepared according to PRISMA 2020 flow diagram [65].

**Fig 6:**
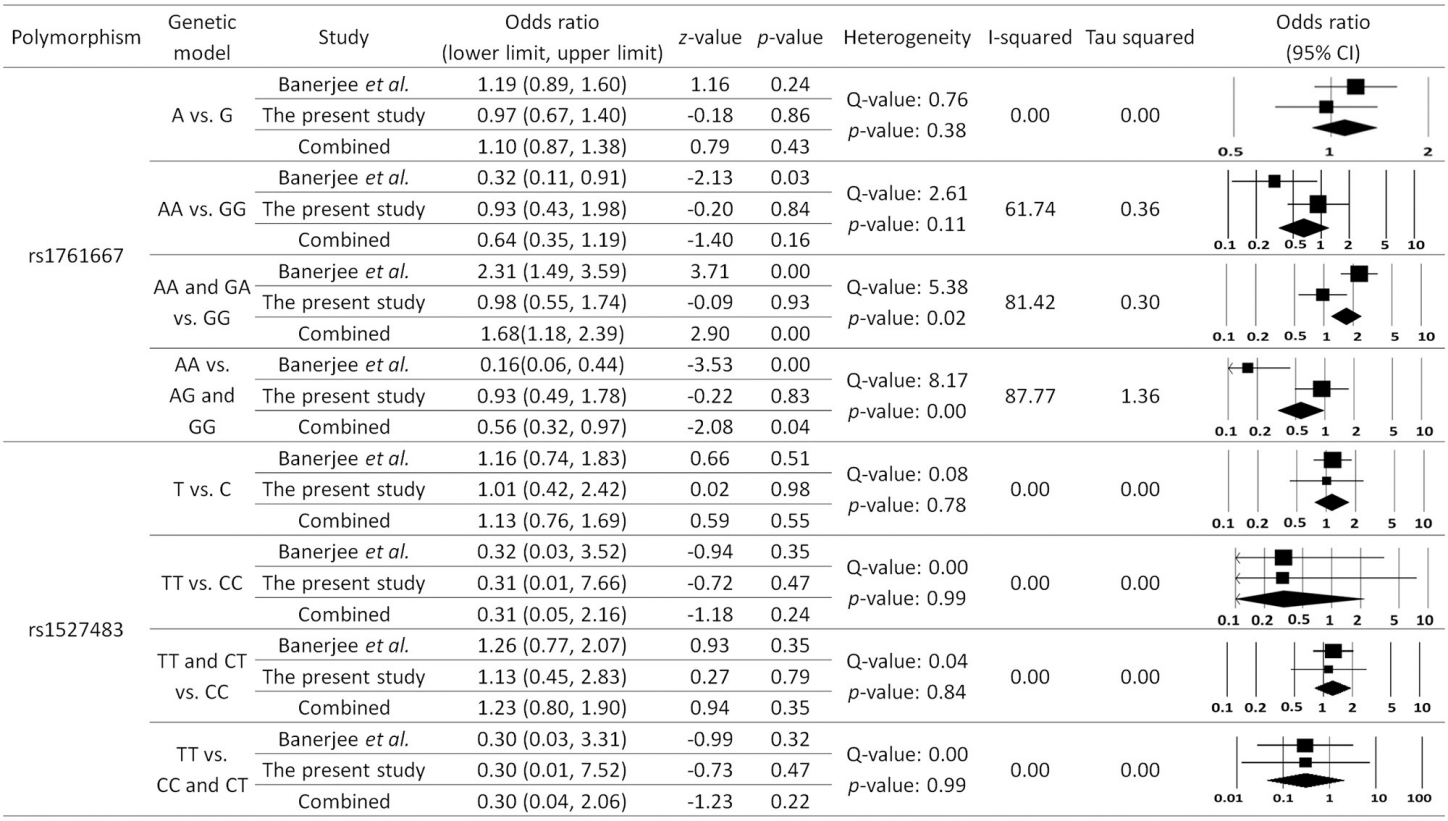
Meta-analysis for both polymorphisms in T2DM group compares to control group. Random effect model is used with all analyses. Based on different genetic models, the frequencies of different genotypes and alleles were extracted from the two studies (i.e., the present study and Banerjee *et al*., 2010 study [24]) and plugged in the software to do the calculations shown above.

The process of article identification and selection is illustrated in Fig 5. A total of 313 articles were found in different databases. Records after duplicates and those that did not meet the inclusion criteria were removed. Thereafter, only one study was eligible and included in quantitative synthesis (Fig 5). Only one study was included in this meta-analysis for T2DM, where both rs1761667 and rs1527483 polymorphisms were studied. The results of meta-analysis are shown in Fig 6. For *CD36* rs1761667, there was no significant difference in the allele frequencies, while genotypes (AA and GA vs. GG) and (AA vs. AG and GG) in both dominant and recessive models, respectively, were significantly different between T2DM and control group (*p* < 0.05), even that our data shows no significant difference (*p* > 0.05). For the other *CD36* polymorphism (i.e., rs1527483), there was no significant association with T2DM for both alleles and genotypes (*p* > 0.05, Fig 6).

## Discussion

Variations in CD36 can lead to several conditions, such as sensory perception, diabetes, coronary heart disease, and others [21, 66]. The role of CD36 in the pathogenesis and prevention of T2DM and lipid metabolism has widespread concerns. CD36 acts as receptor for a broad range of ligands. Ligands can be of proteinaceous nature like thrombospondin, fibronectin, collagen or amyloid-beta as well as of lipidic nature such as oxidized low-density lipoprotein (OxLDL), anionic phospholipids, long-chain fatty acids and bacterial diacylated lipopeptides. They are generally multivalent and can therefore engage multiple receptors simultaneously, the resulting formation of CD36 clusters initiates signal transduction and internalization of receptor-ligand complexes [12, 67, 68].

Multiple observational studies reported a correlation between *CD36* polymorphisms and T2DM [24, 67, 69]. CD36 is involved in functional and physical interactions with many proteins, for example SRC, peroxisome proliferator-activated receptor gamma (PPARG) and toll-like receptor 4 (TLR4). SRC is one of the key regulators of lipid metabolism and diabetes pathogenesis. After activation, it participates in signaling pathways that control a diverse spectrum of biological activities including gene transcription, immune response, cell adhesion, cell cycle progression, apoptosis, migration, and transformation [70].

**Fig 7:**
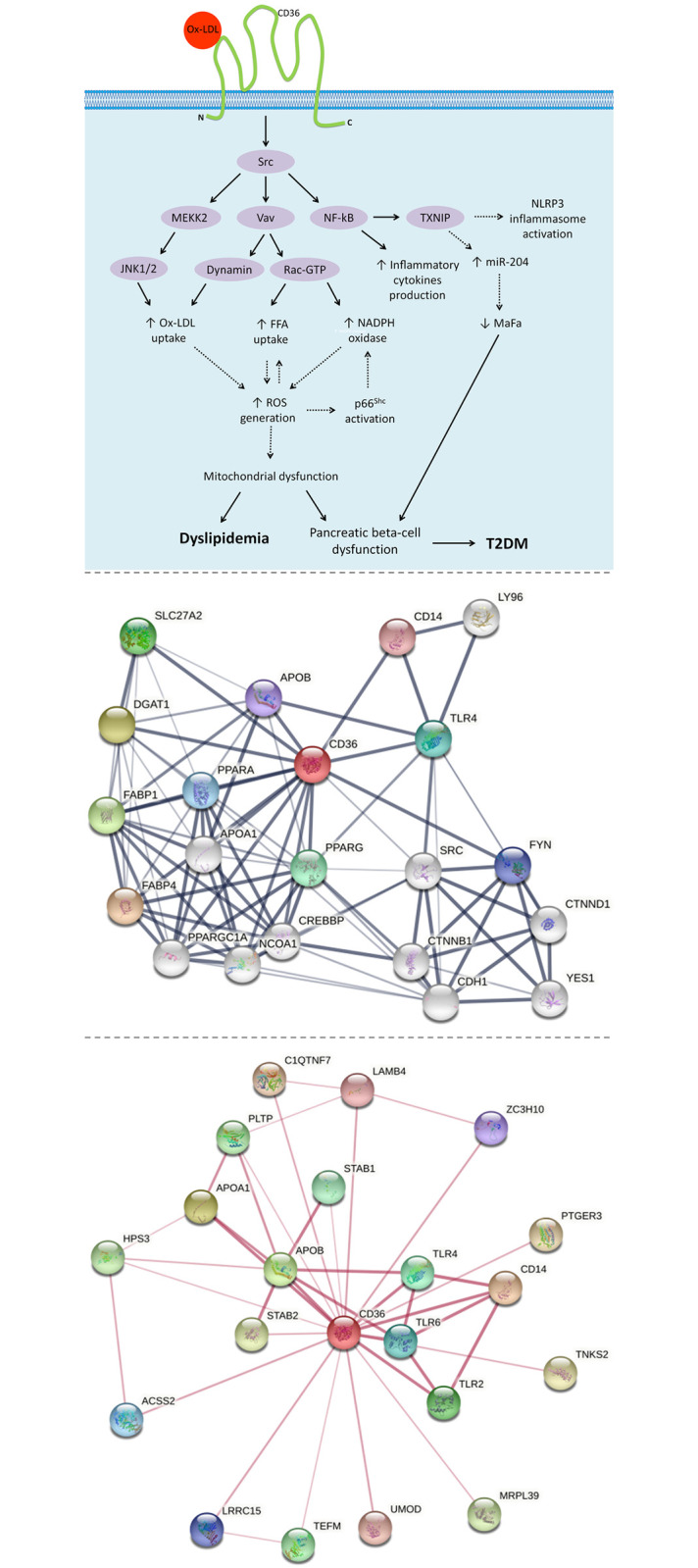
The signal and interaction networks of CD36. The upper illustration represents the potential signaling pathways of Ox-LDL/CD36 that promote T2DM and dyslipidemia. The oxidized low-density lipoprotein (Ox-LDL) initiates the activation of CD36 which bounds to membrane-associated Src family non-receptor tyrosine kinases. This interaction enables three main cytoplasmic signaling domains; nuclear factor kappa B (NF-κB), mitogen-activated protein kinase kinase kinase 2 (MEKK2), and Vav-mediated signaling pathways. Activation of NF-κB contributes to inducing the levels of thioredoxin interacting protein (TXNIP), which in turn activates the NLR family pyrin domain containing 3 (NLRP3) and promotes the production of inflammatory cytokines (e.g., TNF and IL-1). Also, by inducing microRNA (miR-204) expression which targets the insulin transcription factor (MafA), TXNIP contributes to inhibiting insulin production, and thus pancreatic beta-cell dysfunction. Activation of Vav-mediated signaling pathways results in complicated cellular mechanisms finished with increased in the generation of reactive oxygen species (ROS), due to promoting nicotinamide adenine dinucleotide phosphate (NADPH) oxidase activation, free fatty acid (FFA) uptake, and Ox-LDL uptake. Furthermore, through the c-Jun N-terminal protein kinase (JNK1/2) pathway, MEKK2 also promotes Ox-LDL uptake. Thereafter, the excessive generation of ROS causes oxidative damage, and thus results in pancreatic beta-cell dysfunction and evolution of dyslipidemia. The exacerbation of beta-cell dysfunction is involved in the progression of T2DM [71–78]. In the middle illustration, the edges indicate both functional and physical protein associations. Setting included minimum interaction score of 0.15. Max number of interactions is 10 in the first shell and 10 in the second shell. In the lower illustration, the edges indicate that the proteins are part of a physical complex. Setting included minimum interaction score of 0.4. Max number of interactions is 20 in the first shell, and none in the second shell. Line thickness indicates the strength of data support. Both the middle and lower illustrations were created using STRING database.

Fig 7 represents the potential cellular and molecular mechanisms of action for CD36 upon activation by OxLDL, which could be implicated in the development of dyslipidemia and T2DM [71, 72]. Also, for further understanding of CD36 associations to other proteins, a protein-protein interaction network for CD36 is shown in Fig 7.

The purpose of this case-control study was to assess how haplotypes, genotypes and alleles distribution of the *CD36* polymorphisms affects the prevalence of T2DM and dyslipidemia in the Jordanian population. Two different major groups were considered: T2DM patients’ group and the control group. The control group did not deviate from the HWE (*p* > 0.05) (S2 Table).

### Association of *CD36* polymorphisms with T2DM and dyslipidemia

In this comparison, the *CD36* polymorphisms and their respective genotypes were assessed. There were no statistically significant differences for these polymorphisms (p > 0.05) on both T2DM and dyslipidemia. The frequency of the minor allele in the *CD36* polymorphisms was approximately the same in T2DM patients and control subjects. These results fit with those shown by Banerjee *et al*. study for rs1527483, but not rs1761667 [24]. Another study on Egyptian people, involving 100 patients with metabolic syndrome (MS) and 100 control samples showed that the rs1761667 variant was significantly associated with risk of MS [79]. The *CD36* rs1761667 and rs1527483 polymorphisms association results with T2DM and MS in different populations are summarized in Table 12. In this table, the outcome (Yes or No) reveals whether or not there is an association between *CD36* rs1761667 and rs1527483 polymorphisms and T2DM or metabolic syndrome.

**Table 12:** List of studies that have investigated the association of *CD36* polymorphisms (rs1761667 and rs1527483) with T2DM and MS.

Study ID	Country	Ethnicity	Disorder	Cases	Controls	Genotyping	*CD36* SNP	Association
Banerjee et al. [24]	India	Asian	T2DM	250	150	PCR-RFLP	rs1761667	Yes
							rs1527483	No
Bayoumy et al. [79]	Egypt	African	MS	100	100	Allele discrimination technique	rs1761667	Yes
Farook et al. [80]	United States	Mexican American	MS	720	-	Sequencing performed by Polymorphic DNA Technologies	rs1761667	Yes

T2DM, type 2 diabetes mellitus; MS, metabolic syndrome.

### Prediction of diabetes status and dyslipidemia using ML tools

Several ML learners were evaluated against the training and testing set, namely, XGBoost, SVM (C and nu), RF, PNN, NB, kNN, MLP, AdaBoost, and gradient boost. Age, gender, lipid profile, with and without polymorphisms genotypes were evaluated as input descriptors, as indicated in Tables 10 and 11, respectively. Clearly from Table 10, all learners achieved good accuracy; this indicates that the data is self-consistent and predictive. Still, in most cases including polymorphism genotypes didn’t yield apparent better accuracy compared to excluding them, except for Kstar (K*) ML tool. K* can handle noisy data and it requires less time to train the data. However, its performance becomes better with large datasets [81].

Nevertheless, despite the excellent accuracies (i.e., accuracy > 70%) [82] of some ML models (e.g., MLP, RF, and XGBoost) in the present study, to evaluate the behavior of ML tools prompted us to use Cohen’s κ as additional success criteria of the resulting ML models. Cohen’s κ is more robust measure than accuracy, as it takes into account the possibility of prediction by chance [83]. Fleiss’s [84] equally arbitrary guidelines characterize κ over 0.75 as excellent, 0.40 to 0.75 as fair to good, and below 0.40 as poor.

Three learners (C-SVM, nu-SVM, and KNN) failed to yield significant Cohen’s κ values for both 5-fold cross validation and 20% testing set. On the other hand, many of the learners yielded good κ values for 5-fold cross validation but not for the 20% testing set, these ML tools are RF, XGboost, gradient boost, and AdaBoost.

Interestingly, MLP produced good Cohen’s κ scores for both models. Only PNN produced better Cohen’s κ score for the testing set over that 5-fold cross validation. Artificial neural network (MLP) is well-known for its high performance and accuracy. Furthermore, due to the increasing size and complexity of the data, Deep Learning (DL) has been introduced as an improvement to ANN. Recent studies that have used DL produced remarkable results [85, 86].

Moreover, noticeable enhancement in κ score by including rs1761667 and rs1527483 by using K* and AdaBoost, which highlight the importance of such ML tools in the large databases. The best ML tool for predicting T2DM, MLP, was also used to predict dyslipidemia based on FBS, age, gender with and without including rs1761667 and rs1527483 polymorphism genotypes, Despite the fair accuracies (i.e., particularly in 5-fold cross validation), it failed to yield good Cohen’s κ score.

Lai *et al*. used the most recent records of 13,309 Canadian patients aged between 18 and 90 years, along with their demographic and clinical information (age, sex, FBS, body mass index (BMI), HDL, TG, blood pressure (BP) and LDL). Predictive models were built using Logistic Regression and Gradient Boosting Machine (GBM) techniques. They also compared these models to other learning machine techniques such as Decision Tree and RF. According to their findings, The AROC for the proposed GBM model is 84.7% with a sensitivity of 71.6% and the AROC for the proposed Logistic Regression model is 84.0% with a sensitivity of 73.4%. The GBM and Logistic Regression models perform better than the Random Forest and Decision Tree models [87].

Moreover, in Muhammad *et al*. study, the diagnostic dataset of T2DM was collected, and used to develop predictive supervised machine learning models based on logistic regression, SVM, KNN, RF, NB and gradient booting algorithms based on age, family history, glucose, TC, BP, HDL, TG and BMI. The random forest predictive learning-based model appeared to be one of the best developed models with 88.76% in terms of accuracy [44].

**Fig 8:**
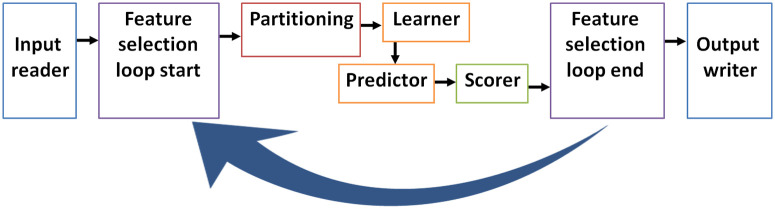
Suggested an efficient platform for medical databases with large number of inputs (features). The platform starts with feeding an input file. Genetic algorithm loop randomly select different subsets (chromosomes) of feature to use them in the prediction process, it starts with "feature selection start" and ends with "feature selection end", and this algorithm saves extensive time in analyzing large databases. Partitioning divides data into training set and testing set, and scorer evaluates the prediction accuracy/Cohen’s κ for the testing set, the results for different chromosomes (subsets of features) can be evaluated. Different Learners and predictors can be selected and evaluated for their performance in prediction (Adapted from Hatmal *et al*. [5]).

Such tools can be implemented in the future for larger databases which include extensive number of cases and input features. In such case, feature selection and weighting tools (i.e., genetic algorithm, SHAP, and stepwise forward and reverse methods) can be implemented to select the best predicting subsets of input features (Fig 8).

### Meta-analysis

The present meta-analysis of CD36 included only one article, in addition to the results of the present study. This meta-analysis was performed under various genetic models, including allelic, homozygous, heterozygous, and dominant models. Random effects meta-analysis has become the standard to combine treatment effects from several studies when the presence of between trial heterogeneity is suspected, which is often the case [88]. In the present meta-analysis, no significant association was found between CD36 rs1527483 and T2DM (*p* > 0.05, Fig 6). For rs1761667, it was found no significant difference in the allele frequencies. However, genotypes (TT, or TT and CT) vs. GG in either dominant or recessive models are significantly different between T2DM and control group (*p* < 0.05), even that our present study had shown no significant difference (*p* > 0.05). The heterogeneity score for these rs1761667 genetic models may represent substantial heterogeneity, which results usually from studies that have confidence intervals (generally depicted graphically using horizontal lines) with poor overlap. This may substantiate performing such meta-analysis on more studies in the future. Meta-analysis of two studies is not uncommon in some diseases, it was concluded that the confidence intervals based on normal quantiles do not have the right coverage and cannot be recommended for use in the case of two studies [88]. While a definite answer to this challenging problem is under dispute, the proposed Bayesian approach works well in many cases. In general, the current methods of meta-analysis have severe limitations, which may be addressed with future research. Until these limitations are resolved, it is recommended to meta-analyze two heterogeneous studies in a Bayesian way using plausible priors [88].

### Limitations and future perspectives

There were some limitations to the current investigation. To begin, a larger sample size of patients and controls may be required to better understand the influence of the *CD36* rs1761667 and rs1527483 polymorphisms on T2DM. Furthermore, machine learning could be used to examine polymorphisms in other *CD36* genotypes and their potential interactions with rs1761667 and rs1527483 variants.

On the other hand, glycated hemoglobin (HbA1c) and other dietary information could be included in the future to assess the relationship between its level and lipid profile. Furthermore, upon implementing these ML tools on a greater number of features, future perspectives could incorporate genetic function algorithms for features reduction and feature importance tools to weight which features significantly contribute to the risk of developing T2DM.

## Materials and methods

### Study design and participants

A total of 350 blood samples (177 samples from T2DM patients, and 173 from subjects with no diabetes) were collected from Jordanian population. To avoid bias in selection of control subjects, they were randomly selected for having no diabetes, but may have other conditions such as obesity, blood pressure and dyslipidemia. Diabetic participants that enrolled in this study were with known history of diabetes and recruited from Jordan University Hospital (JUH). The study was conducted in accordance with the Declaration of Helsinki and was approved by the Institutional Review Board (IRB), JUH, and informed consent form was obtained from each participant.

### Blood samples and chemistry tests

After fasting overnight (8–10 hrs), a total of 10 ml blood (5 ml plain tube, and 5 ml EDTA tube) were collected from every participant. Serum was collected from plain tubes, after centrifugation, and then used to measure the levels of fasting blood sugar (FBS) and lipid profile parameters, including total cholesterol (TC), triglycerides (TG) and HDL by using Cobas C111 analyzer (Roche Diagnostics, Indianapolis, IN, USA). According to Friedewald’s equation [89], the level of LDL was also calculated (Eq 4). Dyslipidemia was defined as having greater than or equal to one of the following conditions: TC ≥ 6.2 mmol/L (240 mg/dL); TG ≥ 2.3 mmol/L (200 mg/dL); HDL ≤ 1.0 mmol/L (40 mg/dL); LDL ≥ 4.1 mmol/L (160 mg/dL) [90].


$$LDL=TC–HDL–\left(\frac{TG}{5}\right)$$ (4)

In the present study, subjects with dyslipidemia were defined based on lipid parameters from both T2DM and control groups. This was done because the diabetes status and FBS were used together with the polymorphisms to predict dyslipidemia.

### DNA extraction, quantification, gel electrophoresis, and sequencing

DNA was extracted from whole-blood samples (EDTA tubes) using the Wizard genomic DNA purification kit (Promega Corporation, Madison, WI, USA), and then a Nano-Drop^™^ 2000/2000c Spectrophotometer (Thermo Fisher Scientific, Waltham, MA, USA) was used to assess the concentration and purity (A260/A280) of the extracted DNA. The extracted DNA were stored at − 20°C until used. Polymerase Chain Reaction (PCR) was used to amplify two CD36 SNPs; rs1527483 (C>T) in intron 11, and rs1761667 (G>A) in the -31118 promoter region of exon 1A. The PCR was performed in a total volume of 25 μl per each reaction containing 50 ng genomic DNA, 5 μl of 5xFIREPol^®^ Master Mix (Solis BioDyne, Tartu, Estonia) and 1 μM of each primer (Gene Link, Hawthorne, NY, USA) by using C1000 Touch^™^ thermal cycler (Bio-Rad, Hercules, CA, USA). The PCR primers and conditions were as described in Table 13.

**Table 13:** Primer sequences for *CD36* polymorphisms, and PCR amplification protocol.

Polymorphism	Primer	Sequence	PCR protocol
rs1527483	F	5’-GCTACAACAATTTTATAGATTTTGAC-’3	Initial denaturation at 95 °C for three min, followed by 35 cycles of 95 °C for 30 sec (denaturation), 60 °C for 40 sec (annealing), and 72 °C for 50 sec (extension), and then a final extension at 72 °C for 10 min.
	R	5’-TGAAATAAAAATAATCTTGTCGATGA-’3	
rs1761667	F	5’-CAAAATCACAATCTATTCAAGACCA-’3	
	R	5’-TTTTGGGAGAAATTCTGAAGAG-’3	

F, forward; R, reverse.

Electrophoresis was used to evaluate PCR amplification, by verifying the migration of DNA fragments in an agarose gel prepared with 1x tis-borate-EDTA (TBE) buffer in a concentration of 2.5% (*m/v*) (2.5 g agarose with 100 mL 1x TBE) and stained with 5.0 μL RedSafe^™^ Nucleic Acid Staining Solution (iNtRON Biotechnology, Seoul, South Korea). Subsequently, 3 μL of the amplified PCR products were loaded and DNA fragments migrated through the gel at 120 Volt for 30 minutes. The gel was then visualized under a UV Transilluminator (UVP Bioimaging System, Upland, CA, USA) to compare the molecular weight of DNA fragments based on a 100 bp DNA ladder.

PCR purification was done by using ExoSAP-IT^™^ kit (Applied Biosystems, Waltham, MA, USA), to eliminate and neutralize PCR residuals, before sending selected samples for DNA sequencing using ABI3730xl DNA Analyzer (Applied Biosystems, Waltham, MA, USA) with big dye terminator version 3.1 kit at Macrogen Inc. (Seoul, South Korea). The determined sequences were aligned with the reference sequence of the *CD36* gene that was downloaded from the NCBI-reference sequences (accession number: NG_008192.1) [91].

### Statistical analysis

The statistical analysis was conducted using SPSS version 16.0 (SPSS Inc., Chicago, IL, USA) and web tool “SNPStats” (www.snpstats.net/analyzer.php) (i.e., odd-ration test, t-test, and chi-square test) [92]. Comprehensive Meta-Analysis (CMA) software package was used for the meta-analysis. In order to verify whether the control group of the present study was under the assumptions of this law, genotype distributions between groups were determined and the Hardy-Weinberg equilibrium (HWE) was carried out.

### Machine learning prediction

For the aim of predicting diabetes or dyslipidemia, several orthogonal ML tools (they use different classification protocols, themes and concepts) were utilized, including MLP (Logistic function), SVM, XGBoost, RF, AdaBoost, gradient boost, PNN, NB and K* were built using version 4.1.3 of KNIME Analytics Platform (KNIME AG, Zurich, Switzerland). Data was used as either 5-fold cross validation, or divided as training set (80%) and testing set (20%). The input-output training set contained the polymorphisms genotypes, gender, age, and clinical parameters (i.e., lipid profile (to predict diabetes) or blood sugar (to predict dyslipidemia)) as inputs, and either the diabetes status (1 for person with diabetes and 0 for person without diabetes) or dyslipidemia status (1 for person with dyslipidemia and 0 for person without dyslipidemia) as output. The clinical parameters used to define the diseases (lipid profile for dyslipidemia, and FBS for T2DM) were excluded from the input data. Herein, we aimed to predict dyslipidemia and T2DM based on other independent factors; no added value if ML tools were used to predict based on the same criteria that were defined by.

#### Random Forest (RF)

RF is a versatile ML approach [34–36], which is based on ensemble of decision trees (DTs), with each tree independently predicting a classification and "voting" for the related class, and the majority of the votes deciding the overall RF predictions [42]. Within the KNIME Analytics Platform, we constructed an RF learner node with the following settings: splitting criterion is the information gain ratio and number of trees (= 100). There were no restrictions on the number of layers or the minimum node size. Out-of-bag internal validation was used to calculate the accuracy.

#### eXtreme gradient boosting (XGBoost)

XGBoost employs an ensemble of weak DT-type models to generate boosted, DT-type models. This system incorporates an unique tree learning algorithm as well as a theoretically justified weighted quantile sketch technique with parallel and distributed computation [41, 42, 93]. We constructed the XGBoost learner node within the KNIME Platform as follows: tree booster was used with depth wise grow policy, boosting rounds = 100, Eta = 0.3, Gamma = 0, maximum depth = 6, minimum child weight = 1, maximum delta step = 0, sub-sampling rate = 1, column sampling rate by tree = 1, column sampling rate by level = 1, lambda = 1, Alpha = 0, sketch epsilon = 0.03, scaled position weight = 1, maximum number of bins = 256, sample type (uniform), normalize type (tree), and dropout rate = 0.

#### k-Nearest Neighbors (kNN)

The kNN classifier is based on a distance learning methodology that calculates an unknown member’s disease status based on the disease status of a set number (k) of nearest neighbors in the training set. A distance metric is used to quantify similarity in this classifier [94]. With k = 6, we implemented the kNN Learner node within the KNIME Analytics Platform.

#### Probabilistic Neural Network (PNN)

PNN is based on the DDA (Dynamic Decay Adjustment) method on labeled data using Constructive Training of Probabilistic Neural Networks. This algorithm generates rules based on numeric data. Each rule is defined as high-dimensional Gaussian function that is adjusted by two thresholds, theta minus and theta plus, to avoid conflicts with rules of different classes [95, 96]. We implemented PNN Learner node within KNIME Analytics Platform using PNN theta minus = 0.2 and theta plus = 0.4 and without specifying maximum number epochs so that the process is repeated until stable rule model is achieved.

#### Naïve Bayesian (NB)

NB is a simple classifier that predicts and assigns class labels to external data based on vectors of descriptors for a finite set of training observations. The NB classifier posits that each descriptor contributes independently to the probability that an observation belongs to a specific class (e.g., disease or no disease) [37–40]. The chance of an observation belonging to a specific class is calculated by multiplying the individual probabilities of that class within each individual descriptor [37–40]. We implemented NB learner node within KNIME Analytics Platform with the following parameters: default probability = 0.0001, minimum standard deviation = 0.0001, threshold standard deviation = 0.0 and maximum number of unique nominal values per attribute = 20.

#### Multilayer Perceptron (MLP)

MLP is a multilayer feed forward network with implementation of the RProp algorithm [97]. MLP is capable of learning nonlinear models in real time. Between the input and output layers of an MLP, one or more nonlinear hidden layers can exist. A varied number of hidden neurons can be allocated to each hidden layer. Each hidden neuron computes a weighted linear sum of the previous layer’s values, and the nonlinear activation function is used. After the output layer transforms the values from the previous hidden layer, the output values are reported. We implemented MLP learner node within KNIME Analytics Platform with the following optimized parameters: Maximum number of iterations = 100, Number of hidden layers = 3, and number of hidden neurons per layer = 100.

#### Support Vector Machine (SVM)

The SVM selects a small number of boundary instances known as support vectors to generate a discriminating function that divides training observations into discrete classes with the broadest possible boundaries. SVM enables the efficient use of a number of kernels for classification. The aim to minimize error on training data and reduce model computational complexity to avoid overfitting by tuning the factors involved in the process is a major characteristic of SVMs [45, 47, 48]. C-SVM and nu-SVM were the two SVM methods tried. The regularization parameters C and nu penalize misclassifications. C ranges from 0 to infinity, while nu ranges from 0 to 1 and indicates the lower and upper bounds on the number of support vector examples that are on the wrong side of the hyper-plane. In both SVM techniques implemented in the WEKA-KNIME LibSVM node, the following default parameters were used: kernel cache (cache size = 40.0), kernel type is radial basis function: exp(-gamma*|u-v|2), loss function is 0.1, kernel coefficients epsilon = 0.001 and Gamma = 0.00. In nu-SVM, however, the optimized nu value of 0.1 was employed.

#### K-star (K*)

K* is an instance-based classifier, it is distinguished from other instance-based learners by its use of an entropy-based distance function. In this learner, the class of a test instance is determined by the class of training instances that are similar to it, as determined by similarity function [98]. The following default settings were used: manual blend setting is 20% and average column entropy curve was used for missing mode.

#### Gradient-boost

The algorithm uses very shallow regression trees and a special form of boosting to build an ensemble of trees. The used base learner for this ensemble method is a simple regression tree as it is used in the tree ensemble, RF and simple regression tree nodes. Per default, a tree is build using binary splits for numeric and nominal attributes [99]. The following default settings were used: tree depth is 4, number of models is 100, and learning rate is 0.1.

#### Adaptive boosting (AdaBoost)

The constructed classifier is composed of multiple weaker models that are independently trained and whose predictions are combined to make the overall prediction [100]. AdaBoost is adaptive in the sense that subsequent weak learners are tweaked in favor of those instances misclassified by previous classifiers. It is likely less susceptible to the overfitting problem than other learning algorithms. The final model can be proven to converge to a strong learner [101]. The following settings were used: percentage of weight mass to base training is set to 100, use resampling for boosting is set as "false", random number seed is 1, and number of iterations is 10.

#### ML model evaluation

The ML models were evaluated by calculating their accuracies (Eq 5) and Cohen’s kappa(κ) values (Eq 6) [5] against the training and testing datasets.

$$Accuracy=\frac{TP+TN}{N}$$ (5)

Where TP represents the true positive, TN represents true negatives, and N represents the total number of cases. Section 3.2.2 contains more information on how it is calculated.

$$K=\frac{P_{0}+P_{e}}{1-P_{e}}$$ (6)

Where P_0_ denotes the observed relative agreement among raters (i.e., accuracy) and P_e_ is the hypothetical probability of random agreement. This is accomplished by calculating the probability of each observer randomly seeing each category based on the observed data. If the raters are completely in agreement, then Cohen’s = 1. Cohen’s = 0 if there is no agreement among the raters other than what would be expected by chance (as given by P_e_). A negative Cohen’s value indicates that the agreement is poorer than random [102].

Training against the training set (80% of data, randomly selected) or 5-fold cross validation of the data points is used for evaluation. The model is then used for classifying the testing data. In 5-fold cross validation, the process is repeated until all training data points are removed from the training list and predicted at least once. Evaluation against the testing set involves calculating the accuracy of the particular ML model by comparing its classification results with the actual disease status of the testing set [103, 104].

### Meta-analysis

**Fig 9:**
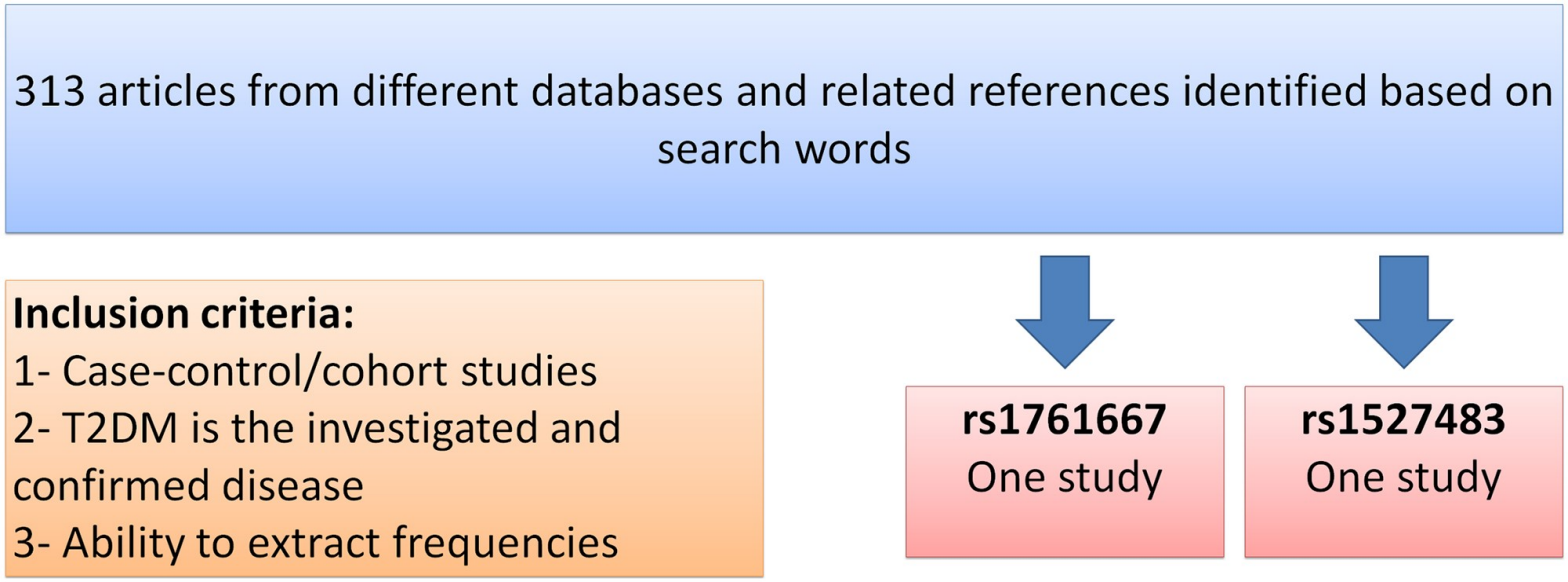
Meta-analysis flow chart and inclusion criteria.

The preferred reporting items for systematic reviews and meta-analyses (PRISMA) guidelines were followed for this work [105]. Literature search was carried out within PubMed (Medline), Google Scholar and Science Direct databases up to February, 2021, using the keywords CD36, gene, patient, polymorphism and disease name (i.e., T2DM/diabetes or dyslipidemia/lipids). Then, potentially relevant publications and studies were retrieved by examining their titles and abstracts and matching the eligible criteria. To facilitate the proper interpretation of results and to minimize heterogeneity, all eligible studies had to fulfill the following inclusion criteria like evaluation of *CD36* gene rs1761667 G>A and rs1527483 C>T with T2DM risk; use of case control or cohort studies; recruitment of pathologically confirmed patients/condition and control subjects; and availability of genotypic frequency both in case and control (Fig 9). The major reasons for exclusion of studies were overlapping data, case only studies, review articles, family-based studies and animal studies. Three authors (M.M.H., M.A.I.A.-H. and O.A.) independently assessed the studies. Studies were included if there was a consensus between the two reviewers.

In addition to the present study, only one study was included in this meta-analysis for T2DM, where both rs1761667 and rs1527483 polymorphisms were studied by PCR-RFLP; that study was conducted by Banerjee *et al*. in North India, and included 250 T2DM cases and 150 healthy controls (all of them from Asian ethnicity) [24]. After extensive search, unfortunately no other studies fit with the inclusion criteria were found on dyslipidemia. The genotypic and allelic frequencies of both polymorphisms for both studies involved in the meta-analysis are shown in S9 Table. Random effect model is used with all analyses. The greatest benefit of conducting the current meta-analysis is to examine sources of heterogeneity, if present, among studies. To the best of our knowledge, there are previous meta-analyses in the literature which covered these *CD36* gene polymorphisms (i.e., rs1761667 and rs1527483), or even other polymorphisms on the *CD36* gene.

## Conclusions

This study has investigated *CD36* gene status in Jordanian subjects by screening for the certain rs1761667 and rs1527483 polymorphisms in T2DM patients compared to control subjects. For both polymorphisms, there was no statistically significant difference between patients and control subjects. However, ML tools (i.e., Logistic, Random Forest, XGBoost, PNN, C-LibSVM, nu-LibSVM, AdaBoost, kNN, K*, and NB) were used as computational platforms to predict subjects with diabetes or dyslipidemia (as output) based on their genotyping results, clinical parameters and demographic data (as input features). Some of these tools had shown high prediction accuracy. Interestingly, in some ML tools (i.e., K*), the prediction accuracy and Cohen’s κ were enhanced by including the genotyping results as inputs. Some ML tools like MLP gave good accuracy and Cohen’s κ in all cases. Indeed, our findings emphasize the importance of embedding ML tools into large medical databases, as well as the potential to forecast patient vulnerability to certain diseases. ML tools can be deployed in medical databases and expanded in the future to include other clinical and genetic parameters, assisting in the early detection of diabetes.

## Supporting information

S1 Table Full details of all people involved in the study. (DOCX)

S2 Table Genotypic and allelic frequencies, and the exact tests for Hardy-Weinberg equilibrium (*n* = 309). (DOCX)

S3 Table Polymorphism rs1761667 and gender cross-classification interaction table. (DOCX)

S4 Table Polymorphism rs1527483 and gender cross-classification interaction table. (DOCX)

S5 Table Haplotype frequencies estimation (*n* = 350). (DOCX)

S6 Table Haplotype and gender cross-classification interaction. (DOCX)

S7 Table Polymorphism rs1761667 and gender cross-classification interaction table. (DOCX)

S8 Table Polymorphism rs1527483 and gender cross-classification interaction. (DOCX)

S9 Table Frequencies and numbers (in brackets) of alleles and genotypes of both polymorphisms for all studies involved in meta-analysis. (DOCX)

S10 Table PRISMA 2020 checklist. (DOCX)

S11 Table Meta-analysis of genetic association studies checklist. (DOCX)
