# Supplementary material for: Investigating the association of CD36 gene polymorphisms (rs1761667 and rs1527483) with T2DM and dyslipidemia: Statistical analysis, machine learning based prediction, and meta-analysis
Source: PLoS One. 2021 Oct 14;16(10):e0257857. doi: 10.1371/journal.pone.0257857 (PMC8516279; doi:10.1371/journal.pone.0257857)
Supplement: S1 Table — (DOCX) [file pone.0257857.s001.docx]

| **S1 Table.** Full details of all participants involved in the study. | | | | | | | | | | | | |
| --- | --- | --- | --- | --- | --- | --- | --- | --- | --- | --- | --- | --- |
| **Sample no.** | **TG** | **LDL** | **HDL** | **TC** | **FBS** | **Gender** | | **Age** | **SNP-1** | **SNP-2** | **T2DM** | **DYS** |
| 1 | 266 | 66.8 | 30 | 150 | 228 | | F | 46 | GA | CC | Y | Y |
| 2 | 273 | 55.4 | 49 | 159 | 155 | | M | 64 | GG | CC | Y | Y |
| 3 | 213 | 105.4 | 32 | 180 | 201 | | M | 65 | GA | CC | Y | Y |
| 4 | 166 | 179.8 | 43 | 256 | 257 | | F | 51 | GA | CC | Y | Y |
| 5 | 196 | 59.8 | 48 | 147 | 149 | | F | 65 | GA | CC | Y | N |
| 6 | 104 | 239.2 | 45 | 305 | 233 | | M | 58 | GG | CT | Y | Y |
| 7 | 253 | 72.4 | 49 | 172 | 89 | | F | 54 | AA | CC | Y | Y |
| 8 | 390 | 121 | 35 | 234 | 297 | | F | 65 | GG | CC | Y | Y |
| 9 | 197 | 53.6 | 25 | 117 | 147 | | M | 60 | GA | CC | Y | Y |
| 10 | 73 | 72.4 | 53 | 140 | 249 | | F | 55 | GA | CC | Y | N |
| 11 | 250 | 29 | 41 | 120 | 393 | | F | 60 | GA | CC | Y | Y |
| 12 | 77 | 143.6 | 49 | 208 | 84 | | M | 50 | GA | CC | Y | N |
| 13 | 140 | 57 | 52 | 137 | 24 | | F | 60 | GG | CC | Y | N |
| 14 | 572 | 63.6 | 38 | 216 | 167 | | F | 68 | GG | CC | Y | Y |
| 15 | 63 | 48.4 | 31 | 92 | 310 | | F | 56 | AA | CT | Y | Y |
| 16 | 199 | 97.2 | 38 | 175 | 237 | | NA | NA | GG | CC | Y | Y |
| 17 | 135 | 43 | 69 | 139 | 173 | | NA | 57 | GG | CC | Y | N |
| 18 | 268 | 26 | 116.4 | 256 | 395 | | NA | 45 | GA | CC | Y | Y |
| 19 | 149 | 28 | 82 | 140 | 235 | | NA | 70 | GA | CC | Y | N |
| 20 | 118 | 42 | 43 | 109 | 242 | | NA | 65 | AA | CC | Y | N |
| 21 | 237 | 28 | 78 | 154 | 170 | | NA | 65 | GA | CC | Y | Y |
| 22 | 133 | 39 | 128 | 190 | 249 | | NA | 38 | GG | CC | Y | N |
| 23 | 70 | 53 | 37 | 104 | 401 | | NA | 39 | GA | CC | Y | Y |
| 24 | 96 | 47 | 105 | 171 | 203 | | NA | 72 | GA | CC | Y | N |
| 25 | 155 | 28 | 99 | 158 | 226 | | NA | 50 | GA | CC | Y | N |
| 26 | 140 | 23 | 86 | 137 | 126 | | NA | 63 | GA | CC | Y | N |
| 27 | 142 | 151.6 | 29 | 209 | 198 | | M | 75 | GG | CC | Y | Y |
| 28 | 202 | 73.6 | 27 | 141 | 370 | | F | 42 | GG | CC | Y | Y |
| 29 | 171 | 103.8 | 23 | 161 | 482 | | F | 66 | AA | CC | Y | Y |
| 30 | 115 | 163 | 45 | 231 | 170 | | F | 60 | GA | CC | Y | Y |
| 31 | 138 | 150.4 | 56 | 234 | 410 | | F | 33 | GA | CC | Y | N |
| 32 | 230 | 178 | 22 | 246 | 278 | | M | 65 | GA | CC | Y | Y |
| 33 | 306 | NA | 18 | 169 | 309 | | M | 52 | GG | CC | Y | Y |
| 34 | 160 | 118 | 51 | 201 | 122 | | M | 80 | AA | CC | Y | N |
| 35 | 213 | 134.4 | 29 | 206 | 321 | | F | 54 | GA | CC | Y | Y |
| 36 | 306 | NA | 16 | 213 | 144 | | M | 60 | GA | CC | Y | Y |
| 37 | 154 | 52.2 | 36 | 119 | 185 | | F | 67 | GA | CC | Y | Y |
| 38 | 133 | 149.4 | 28 | 204 | 268 | | F | 51 | GA | CC | Y | Y |
| 39 | 200 | 127 | 31 | 198 | 334 | | F | 47 | GA | CC | Y | Y |
| 40 | 248 | 121.4 | 36 | 207 | 405 | | F | 54 | GG | CC | Y | Y |
| 41 | 722 | NA | 14 | 179 | 258 | | F | 35 | GA | CT | Y | Y |
| 42 | 294 | 40.2 | 41 | 140 | 270 | | F | 50 | AA | CC | Y | Y |
| 43 | 294 | 39.2 | 16 | 114 | 195 | | M | 66 | GA | CC | Y | Y |
| 44 | 142 | 60.6 | 25 | 114 | 230 | | M | 57 | GA | CC | Y | Y |
| 45 | 250 | 23 | 26 | 99 | 197 | | F | 50 | GA | CC | Y | Y |
| 46 | 276 | NA | 18 | 196 | 110 | | M | 72 | GG | CC | Y | Y |
| 47 | 290 | 59 | 26 | 143 | 270 | | F | 59 | GA | CC | Y | Y |
| 48 | 484 | NA | 23 | 160 | 201 | | M | 52 | GG | CC | Y | Y |
| 49 | 108 | 49.4 | 26 | 97 | 158 | | F | 52 | GA | CC | Y | Y |
| 50 | 338 | NA | 26 | 111 | 230 | | M | 37 | AA | CC | Y | Y |
| 51 | 200 | 133 | 23 | 196 | 114 | | F | 48 | GA | CC | Y | Y |
| 52 | 372 | 81.6 | 29 | 185 | 219 | | F | 49 | GG | CC | Y | Y |
| 53 | 266 | 73.8 | 27 | 154 | 390 | | F | 57 | GA | CC | Y | Y |
| 54 | 296 | 150.8 | 24 | 234 | 158 | | F | 45 | GA | CC | Y | Y |
| 55 | 224 | 142.2 | 27 | 214 | 280 | | F | 68 | GA | CT | Y | Y |
| 56 | 234 | 131.2 | 38 | 216 | 283 | | M | 43 | AA | CC | Y | Y |
| 57 | 264 | 7.2 | 34 | 94 | 315 | | F | 68 | GG | CC | Y | Y |
| 58 | 142 | 198.6 | 37 | 264 | 129 | | F | 44 | GA | CC | Y | Y |
| 59 | 230 | 145 | 21 | 230 | 274 | | F | 50 | GA | CC | Y | Y |
| 60 | 272 | 117.6 | 33 | 205 | 351 | | F | 46 | GA | CC | Y | Y |
| 61 | 186 | 45.8 | 22 | 105 | 170 | | F | 52 | GG | CC | Y | Y |
| 62 | 187 | 100.4 | 36 | 187 | 141 | | F | 54 | GA | CC | Y | Y |
| 63 | 178 | 65.2 | 24.2 | 125 | 348 | | M | 66 | AA | CC | Y | Y |
| 64 | 295 | 94.1 | 24.9 | 178 | 174 | | F | 50 | GA | CC | Y | Y |
| 65 | 181 | 66.6 | 24.2 | 177 | 194 | | M | 57 | GG | CC | Y | Y |
| 66 | 230 | 6 | 55 | 107 | 165 | | F | 59 | GA | CC | Y | Y |
| 67 | 331 | 1.37 | 28.2 | 232 | 123 | | M | 52 | AA | CC | Y | Y |
| 68 | 226 | 61.7 | 43.9 | 151 | 136 | | F | 52 | GA | CC | Y | Y |
| 69 | 411 | 1.6 | 28.2 | 112 | 122 | | M | 37 | GG | CC | Y | Y |
| 70 | 142 | NA | NA | 195 | 225 | | F | 50 | GA | CC | Y | Y |
| 71 | 77 | 94.8 | 49.8 | 160 | 139 | | M | 72 | AA | CC | Y | N |
| 72 | 174 | 73.6 | 42.6 | 151 | 205 | | F | 48 | GA | CC | Y | N |
| 73 | NA | NA | NA | 191 | 225 | | M | 55 | GA | CC | Y | Y |
| 74 | NA | NA | NA | 109 | 182 | | F | 51 | GG | CC | Y | Y |
| 75 | 355 | 5 | 34 | 110 | 236 | | M | 50 | AA | CC | Y | Y |
| 76 | NA | NA | NA | NA | NA | | NA | NA | GA | CC | Y | Y |
| 77 | NA | NA | NA | 193 | 162 | | F | 71 | GG | CC | Y | Y |
| 78 | NA | NA | NA | 208 | 303 | | F | 69 | GA | CC | Y | Y |
| 79 | NA | NA | NA | 269 | 106 | | F | 76 | GG | CC | Y | Y |
| 80 | NA | NA | NA | 214 | 220 | | F | 66 | GA | CC | Y | Y |
| 81 | 366 | 36.8 | 24 | 134 | 209 | | M | 42 | GA | CC | Y | Y |
| 82 | 271 | 116.8 | 30 | 201 | 119 | | M | 58 | AA | CC | Y | Y |
| 83 | 310 | 104 | 28 | 194 | 147 | | F | 55 | GG | CC | Y | Y |
| 84 | 168 | 83.4 | 50 | 167 | 273 | | F | 54 | GA | CC | Y | N |
| 85 | 355 | 83 | 33 | 187 | 262 | | M | 57 | GA | CC | Y | Y |
| 86 | 168 | 135.4 | 58 | 227 | 135 | | F | 48 | GA | CT | Y | N |
| 87 | 133 | 61.4 | 34 | 122 | 433 | | M | 42 | GG | CC | Y | Y |
| 88 | 90 | 116 | 31 | 165 | 355 | | F | 54 | AA | CC | Y | Y |
| 89 | 228 | 12.04 | 41 | 207 | 205 | | F | 20 | GA | CC | Y | Y |
| 90 | 353 | NA | 37 | 154 | 412 | | F | 62 | NA | CC | Y | Y |
| 91 | 81 | NA | 50 | 162 | 138 | | F | 41 | GG | CC | Y | Y |
| 92 | 320 | 61.8 | 69 | 272 | 181 | | F | 60 | GG | CC | Y | Y |
| 93 | 265 | 44.8 | 54 | 191 | 275 | | M | 53 | AA | CC | Y | Y |
| 94 | 333 | NA | 40 | 278 | 276 | | F | 45 | GG | CC | Y | Y |
| 95 | 391 | 51 | NA | 177 | 475 | | F | 55 | GA | CC | Y | Y |
| 96 | 187 | 58.6 | 35 | 131 | 155 | | M | 50 | GG | CC | Y | Y |
| 97 | 170 | 94 | 47 | 175 | 201 | | F | 53 | GG | CT | Y | N |
| 98 | 237 | 14.6 | 53 | 115 | 195 | | F | 75 | GG | CC | Y | Y |
| 99 | 510 | 94 | 24 | 220 | 390 | | F | 65 | GA | CC | Y | Y |
| 100 | 178 | 151.4 | 20 | 207 | 329 | | F | 55 | AA | CC | Y | Y |
| 101 | 310 | 104 | 28 | 194 | 147 | | F | 55 | NA | CC | Y | Y |
| 102 | NA | NA | NA | 156 | 158 | | F | 48 | NA | CC | Y | Y |
| 103 | NA | NA | NA | 230 | 131 | | M | 72 | NA | CC | Y | Y |
| 104 | NA | NA | NA | 168 | 209 | | F | 72 | AA | CC | Y | Y |
| 105 | 111 | 122 | 56 | 200 | 127 | | M | 57 | NA | CC | Y | N |
| 106 | 242 | 127 | 30 | 178 | NA | | M | 72 | NA | CC | Y | Y |
| 107 | NA | NA | NA | NA | NA | | M | 45 | NA | CC | Y | Y |
| 108 | 150 | 154 | 44 | 228 | 105 | | F | 46 | GA | CC | Y | N |
| 109 | 147 | 105 | 51 | 185 | 177 | | F | 76 | GG | CC | Y | N |
| 110 | 156 | 116 | 24 | 171 | 126 | | M | 57 | GA | CC | Y | Y |
| 111 | 155 | 65 | 67 | 155 | 209 | | F | 45 | GA | CC | Y | N |
| 112 | 315 | 108 | 31 | 179 | 154 | | F | 70 | GA | CC | Y | Y |
| 113 | 164 | 85 | 39 | 157 | NA | | M | 62 | GA | CC | Y | Y |
| 114 | 306 | 120 | 45 | 226 | 154 | | F | 60 | GG | CC | Y | Y |
| 115 | 174 | 100 | 53 | 173 | 208 | | F | 64 | NA | CC | Y | N |
| 116 | 147 | 147 | 47 | 223 | NA | | M | 51 | GA | CC | Y | N |
| 117 | 112 | 95 | 67 | 184 | 111 | | F | 72 | GA | CC | Y | N |
| 118 | 265 | 137 | 35 | 225 | 112 | | M | 45 | GA | CT | Y | Y |
| 119 | 128 | 127 | 43 | 196 | 162 | | M | 70 | AA | CC | Y | N |
| 120 | 421 | 158 | 29 | 238 | NA | | M | 42 | GG | CC | Y | Y |
| 121 | 436 | 116 | NA | 194 | 154 | | M | 59 | AA | CC | Y | Y |
| 122 | 231 | 151 | 43 | 240 | 128 | | F | 55 | GA | CC | Y | Y |
| 123 | 268 | 100 | 42 | 201 | 141 | | M | 40 | GA | CC | Y | Y |
| 124 | 233 | 111 | 31 | 189 | 189 | | M | 69 | GG | CT | Y | Y |
| 125 | 124 | 42 | 58 | 195 | 237 | | M | 47 | GA | CC | Y | N |
| 126 | 227 | 41 | 96 | 128 | NA | | M | 47 | GG | CC | Y | Y |
| 127 | 277 | 174 | 41 | 270 | 229 | | M | 57 | GA | CC | Y | Y |
| 128 | 166 | 153 | 46 | 232 | 82 | | F | 44 | AA | CC | Y | N |
| 129 | 151 | 104 | 35 | 169 | NA | | F | 48 | GG | CC | Y | Y |
| 130 | 237 | 175 | 38 | 260 | 126 | | M | 67 | AA | CC | Y | Y |
| 131 | 144 | 74 | 54 | 159 | 128 | | F | 71 | GG | CC | Y | N |
| 132 | 192 | 73 | 47 | 159 | 216 | | F | 59 | GA | CC | Y | N |
| 133 | 163 | 43 | 36 | 162 | 125 | | M | 53 | GG | CC | Y | Y |
| 134 | 340 | 204 | 34 | 274 | 93 | | M | 44 | GG | CT | Y | Y |
| 135 | 148 | 65 | 35 | 130 | 112 | | M | 70 | GG | CC | Y | Y |
| 136 | 116 | 134 | 50 | 207 | 111 | | M | 44 | AA | CC | Y | N |
| 137 | 191 | 114 | 36 | 189 | 164 | | F | 73 | GG | CC | Y | Y |
| 138 | 315 | 115 | 39 | 217 | 129 | | M | 49 | GA | CC | Y | Y |
| 139 | 89 | 94 | 84 | 196 | 143 | | M | 68 | GG | CC | Y | N |
| 140 | 83 | 86 | 53 | 156 | NA | | M | 54 | GG | CC | Y | N |
| 141 | 403 | 158 | 35 | 227 | 127 | | M | 51 | GA | CC | Y | Y |
| 142 | 138 | 69 | 39 | 136 | 113 | | M | 61 | GA | CC | Y | Y |
| 143 | 402 | 125 | 125 | 201 | 296 | | F | 34 | GA | CC | Y | Y |
| 144 | 81 | 106 | 51 | 173 | 144 | | M | 89 | AA | CC | Y | N |
| 145 | 139 | 76 | 40 | 144 | 155 | | F | 51 | AA | CC | Y | Y |
| 146 | 249 | 55 | 35 | 113 | 110 | | M | 73 | GA | CC | Y | Y |
| 147 | 182 | 53 | 32 | 121 | 137 | | F | 70 | GG | CC | Y | Y |
| 148 | 230 | 81 | 34 | 161 | NA | | M | 52 | AA | CC | Y | Y |
| 149 | 95 | 85 | 34 | 138 | 130 | | F | 67 | AA | CC | Y | Y |
| 150 | 148 | 177 | 54 | 261 | 128 | | F | 78 | GA | CC | Y | Y |
| 151 | 134 | 55 | 40 | 122 | 99 | | F | 53 | GA | CC | Y | Y |
| 152 | 118 | 194 | 42 | 260 | 260 | | M | 46 | AA | CC | Y | Y |
| 153 | 136 | 77 | 53 | 157 | 152 | | F | 55 | NA | CT | Y | N |
| 154 | 218 | 62 | 44 | 150 | 151 | | M | 46 | GA | CC | Y | Y |
| 155 | 116 | 142 | 41 | 206 | 122 | | M | 82 | GA | CC | Y | N |
| 156 | 137 | 138 | 54 | 219 | 160 | | M | 65 | AA | CC | Y | N |
| 157 | 185 | 185 | 130 | 56 | 116 | | M | 69 | GG | CC | Y | Y |
| 158 | 90 | 65 | 40 | 123 | 167 | | M | 67 | GA | CT | Y | Y |
| 159 | 211 | 124 | 33 | 199 | 137 | | M | 57 | GA | CC | Y | Y |
| 160 | 150 | 73 | 57 | 160 | 167 | | F | 71 | GG | CC | Y | N |
| 161 | 211 | 112 | 48 | 202 | 336 | | F | 59 | GA | CC | Y | Y |
| 162 | 84 | 68 | 41 | 127 | 108 | | M | 61 | GA | CC | Y | N |
| 163 | 241 | 98 | 29 | 175 | 135 | | F | 67 | GA | CC | Y | Y |
| 164 | 147 | 73 | 76 | 178 | 107 | | F | 72 | GG | CC | Y | N |
| 165 | 254 | 163 | 44 | 258 | NA | | F | 79 | AA | CC | Y | Y |
| 166 | 342 | 171 | 33 | 274 | 92 | | F | 81 | GA | CC | Y | Y |
| 167 | 153 | 157 | 37 | 212 | 180 | | F | 52 | GG | CC | Y | Y |
| 168 | 248 | 102 | 49 | 201 | 193 | | F | 78 | GG | CT | Y | Y |
| 169 | 86 | 82 | 68 | 167 | 183 | | F | 73 | AA | CC | Y | N |
| 170 | 165 | 151 | 45 | 229 | 105 | | F | 64 | GA | CC | Y | N |
| 171 | 218 | 18 | 42 | 102 | 109 | | F | 41 | GG | CC | Y | Y |
| 172 | 122 | 126 | 43 | 193 | 199 | | M | 73 | GA | CC | Y | N |
| 173 | 139 | 57 | 178 | 102 | 137 | | M | 71 | GA | CC | Y | N |
| 174 | 136 | 119 | 42 | 188 | 195 | | F | 60 | AA | CC | Y | N |
| 175 | 99 | 52 | 36 | 108 | 109 | | M | 41 | GG | CC | Y | Y |
| 176 | 158 | 98 | 34 | 163 | 129 | | M | 46 | GA | CC | Y | Y |
| 177 | 121 | 103 | 34 | 165 | 152 | | M | 53 | GA | CC | Y | Y |
| 178 | 51 | 85 | NA | 149 | 89 | | M | 35 | GA | CC | N | N |
| 179 | 76 | 86 | 38 | 139 | 102 | | M | 61 | GG | CC | N | Y |
| 180 | 90 | 93 | 51 | 162 | 103 | | M | 58 | GA | CC | N | N |
| 181 | 119 | 96 | 49 | 166 | 89 | | M | 72 | GA | CC | N | N |
| 182 | 94 | 155 | 42 | 216 | NA | | M | 44 | AA | CT | N | N |
| 183 | 105 | 140 | 401 | 201 | NA | | M | 53 | GA | CC | N | N |
| 184 | 557 | 152 | 32 | 295 | 132 | | M | 71 | NA | CC | N | Y |
| 185 | NA | NA | NA | NA | NA | | M | 54 | AA | CC | N | Y |
| 186 | 175 | 148 | 48 | 231 | NA | | M | 66 | NA | CC | N | N |
| 187 | 109 | 108 | 51 | 181 | NA | | F | 47 | GA | CC | N | N |
| 188 | 170 | 82 | 60 | 176 | 96 | | F | 67 | NA | CT | N | N |
| 189 | 125 | NA | 76 | 250 | 92 | | F | 36 | NA | CC | N | Y |
| 190 | 217 | 160 | 36 | 239 | 102 | | F | 42 | GG | CC | N | Y |
| 191 | 113 | 143 | 56 | 222 | 94 | | F | 63 | GA | CC | N | N |
| 192 | 112 | 147 | 34 | 203 | 89 | | M | 68 | AA | CC | N | Y |
| 193 | 102 | 104 | 58 | 182 | 95 | | F | 47 | GA | CC | N | N |
| 194 | 208 | 138 | 37 | 217 | 87 | | M | 62 | AA | CC | N | Y |
| 195 | 226 | 102 | 33 | 184 | 98 | | M | 50 | GA | CC | N | Y |
| 196 | 97 | 124 | 56 | 199 | 91 | | M | NA | AA | CC | N | N |
| 197 | 129 | 125 | 45 | 196 | 99 | | M | 42 | AA | CC | N | N |
| 198 | 135 | 153 | 36 | 216 | 101 | | M | 51 | GA | CC | N | Y |
| 199 | 155 | 108 | 37 | 176 | 95 | | M | 53 | GA | CC | N | Y |
| 200 | 72 | 113 | 75 | 202 | NA | | F | 37 | NA | CC | N | N |
| 201 | 263 | 131 | 35 | 219 | NA | | M | 45 | GA | CC | N | Y |
| 202 | 194 | 144 | 51 | 234 | 95 | | F | 43 | AA | CC | N | N |
| 203 | 119 | 125 | 54 | 203 | 92 | | M | 49 | GA | CC | N | N |
| 204 | 53 | 110 | 56 | 177 | NA | | F | 26 | AA | CC | N | N |
| 205 | 138 | 158 | 37 | 223 | 93 | | M | 51 | GA | CC | N | Y |
| 206 | 37 | 82 | 66 | 155 | NA | | F | 30 | GA | CC | N | N |
| 207 | 89 | 121 | 54 | 193 | 87 | | M | 36 | GG | CC | N | N |
| 208 | 81 | 100 | 54 | 170 | 80 | | M | 20 | GG | CC | N | N |
| 209 | 209 | 142 | 32 | 216 | 103 | | M | 42 | GA | CC | N | Y |
| 210 | 89 | 98 | 53 | 168 | 102 | | M | 58 | GA | CC | N | N |
| 211 | 309 | 83 | 36 | 181 | NA | | M | 38 | GA | CC | N | Y |
| 212 | 123 | 120 | 37 | 182 | 106 | | M | 44 | GA | CC | N | Y |
| 213 | 104 | 88 | 74 | 183 | NA | | F | 56 | GA | CC | N | N |
| 214 | 109 | 106 | 39 | 167 | 104 | | M | 29 | GG | CC | N | Y |
| 215 | 117 | 124 | 55 | 202 | NA | | F | 46 | AA | CC | N | N |
| 216 | 128 | 110 | 54 | 190 | 99 | | M | 54 | GA | CC | N | N |
| 217 | 129 | 105 | 31 | 162 | NA | | M | 65 | NA | CC | N | Y |
| 218 | 155 | 63 | 74 | 168 | NA | | F | 57 | GG | CC | N | N |
| 219 | 71 | 75 | 34 | 123 | 83 | | M | 33 | GG | CC | N | Y |
| 220 | 140 | 101 | 46 | 175 | 91 | | F | 57 | GA | CC | N | N |
| 221 | NA | 112 | 80 | 206 | 92 | | F | 31 | GA | CC | N | Y |
| 222 | 192 | 84 | 43 | 165 | 111 | | M | 44 | GA | CC | N | N |
| 223 | 105 | 130 | 46 | 197 | 115 | | M | 53 | AA | CC | N | N |
| 224 | 112 | 89 | 60 | 171 | 93 | | F | 87 | GA | CC | N | N |
| 225 | 103 | 137 | 45 | 203 | 100 | | M | 61 | GG | CC | N | N |
| 226 | 121 | 138 | 52 | 214 | 86 | | M | 55 | GG | CC | N | N |
| 227 | 59 | 143 | 68 | 227 | NA | | F | 40 | GG | CC | N | N |
| 228 | 63 | 188 | 86 | 287 | 88 | | M | 54 | AA | CC | N | Y |
| 229 | 178 | 110 | 58 | 204 | 100 | | M | 47 | GG | CC | N | N |
| 230 | 221 | 63 | 33 | 140 | 100 | | M | 44 | GG | CC | N | Y |
| 231 | 133 | 157 | 59 | 243 | 99 | | M | 54 | AA | CC | N | Y |
| 232 | 168 | 116 | 41 | 191 | 102 | | F | 35 | GA | CC | N | N |
| 233 | 169 | 116 | 41 | 191 | 102 | | F | 55 | GA | CC | N | N |
| 234 | 91 | 132 | 52 | 202 | 102 | | F | 60 | GG | CC | N | N |
| 235 | 248 | 185 | 25 | 260 | 95 | | M | 35 | GA | CC | N | Y |
| 236 | 163 | 144 | 31 | 208 | 82 | | M | 47 | NA | CC | N | Y |
| 237 | 178 | 97 | 57 | 190 | 94 | | F | 46 | GG | CC | N | N |
| 238 | 218 | 97 | 47 | 188 | 90 | | F | 37 | AA | CC | N | Y |
| 239 | 86 | 144 | 65 | 216 | 91 | | F | 50 | GG | CC | N | N |
| 240 | 96 | 147 | 54 | 220 | NA | | F | 31 | GG | CC | N | N |
| 241 | 106 | 74 | 53 | 147 | 92 | | F | 77 | GA | CC | N | N |
| 242 | 376 | 207 | 31 | 313 | 98 | | M | 54 | GA | CC | N | Y |
| 243 | 142 | 132 | 63 | 227 | 110 | | F | 54 | GG | CC | N | N |
| 244 | 177 | 105 | 40 | 181 | NA | | F | 63 | AA | CC | N | Y |
| 245 | 103 | 104 | 63 | 188 | 102 | | F | 41 | GA | CC | N | N |
| 246 | 134 | 85 | 34 | 151 | 92 | | M | 42 | GA | CC | N | Y |
| 247 | 74 | 79 | 47 | 141 | 97 | | F | 57 | GA | CC | N | N |
| 248 | 282 | 139 | 30 | 225 | 123 | | M | 45 | GA | CC | N | Y |
| 249 | 197 | 82 | 47 | 168 | NA | | F | 34 | AA | CC | N | N |
| 250 | 239 | 240 | 44 | 309 | 107 | | F | 62 | GA | CC | N | Y |
| 251 | 71 | 194 | 57 | 270 | 91 | | M | 47 | AA | CC | N | Y |
| 252 | 90 | 185 | 59 | 262 | NA | | F | 46 | GA | CC | N | Y |
| 253 | 69 | 132 | 63 | 209 | 94 | | F | 37 | NA | CT | N | N |
| 254 | 43 | 76 | 92 | 177 | 80 | | F | 24 | NA | CC | N | N |
| 255 | 53 | 92 | 58 | 161 | NA | | M | 61 | NA | CT | N | N |
| 256 | 78 | 156 | 42 | 214 | 116 | | M | 42 | NA | CC | N | N |
| 257 | 138 | 120 | 58 | 204 | 92 | | F | 49 | GA | CC | N | N |
| 258 | 299 | 110 | 31 | 201 | 85 | | M | 36 | NA | CC | N | Y |
| 259 | 147 | 134 | 45 | 208 | 94 | | M | 49 | GA | CC | N | N |
| 260 | 182 | 109 | 31 | 176 | 88 | | M | 29 | GG | CC | N | Y |
| 261 | 146 | 115 | 67 | 211 | 102 | | F | 69 | AA | CC | N | N |
| 262 | 145 | 115 | 44 | 188 | 109 | | M | 55 | NA | CC | N | N |
| 263 | 208 | 141 | 53 | 235 | NA | | F | 58 | NA | CC | N | Y |
| 264 | 151 | 210 | 60 | 300 | 101 | | M | 55 | AA | CC | N | Y |
| 265 | 184 | 141 | 47 | 225 | 101 | | F | 55 | NA | CC | N | N |
| 266 | 161 | 162 | 39 | 233 | 140 | | M | 75 | GA | CC | N | Y |
| 267 | 89 | 100 | 40 | 158 | 92 | | M | 37 | GG | CC | N | Y |
| 268 | 142 | 151 | 47 | 226 | 129 | | F | 70 | GA | CC | N | N |
| 269 | 105 | 112 | 57 | 190 | 97 | | M | 73 | GG | CT | N | N |
| 270 | 66 | 102 | 53 | 168 | 103 | | M | 60 | NA | CC | N | N |
| 271 | 144 | 142 | 36 | 207 | 115 | | F | 68 | GG | CC | N | Y |
| 272 | 102 | 80 | 67 | 195 | NA | | F | 41 | NA | CC | N | N |
| 273 | 41 | 44 | 73 | 125 | 94 | | F | 52 | GA | CC | N | N |
| 274 | 107 | 132 | 53 | 206 | 103 | | M | 55 | GG | TT | N | N |
| 275 | 210 | 118 | 41 | 201 | 94 | | M | 62 | NA | CT | N | Y |
| 276 | 48 | 112 | 58 | 180 | NA | | F | 38 | GA | CC | N | N |
| 277 | 117 | 144 | 61 | 228 | 89 | | F | 59 | AA | CC | N | N |
| 278 | 197 | 113 | 52 | 204 | NA | | F | 49 | NA | CC | N | N |
| 279 | 76 | 113 | 48 | 176 | 92 | | M | NA | GA | CC | N | N |
| 280 | 117 | 76 | 60 | 159 | 91 | | F | NA | NA | CC | N | N |
| 281 | NA | 88 | 121 | 163 | 107 | | M | 41 | GA | CC | N | Y |
| 282 | 117 | 111 | 61 | 193 | 94 | | M | 48 | GA | CC | N | N |
| 283 | 117 | 111 | 61 | 195 | 94 | | M | 48 | GA | CC | N | N |
| 284 | 101 | 125 | 57 | 202 | 107 | | F | 49 | GA | CC | N | N |
| 285 | 262 | 83 | 44 | 179 | NA | | M | 42 | GA | CC | N | Y |
| 286 | 119 | 80 | 51 | 155 | 92 | | F | 34 | NA | CC | N | N |
| 287 | 165 | 110 | 35 | 165 | 95 | | F | 50 | GA | CC | N | Y |
| 288 | 126 | 109 | 46 | 180 | 111 | | F | 68 | GA | CC | N | N |
| 289 | 63 | 127 | 59 | 199 | 86 | | F | 42 | NA | CC | N | N |
| 290 | 88 | 125 | 47 | 190 | 83 | | M | 35 | GA | CC | N | N |
| 291 | 240 | 136 | 28 | 212 | NA | | M | 36 | GA | CC | N | Y |
| 292 | 165 | 110 | 50 | 193 | 100 | | M | 44 | GA | CC | N | N |
| 293 | 233 | 116 | 30 | 193 | 91 | | M | 46 | GA | CC | N | Y |
| 294 | 139 | 79 | 30 | 137 | NA | | M | 55 | NA | CT | N | Y |
| 295 | 178 | 144 | 48 | 228 | NA | | M | 92 | GA | CT | N | N |
| 296 | 73 | 75 | 68 | 158 | NA | | F | 49 | GG | CT | N | N |
| 297 | 121 | 113 | 48 | 185 | 83 | | M | 41 | NA | CT | N | N |
| 298 | 74 | 120 | 62 | 197 | 94 | | M | 45 | NA | CT | N | N |
| 299 | 70 | 90 | 48 | 152 | NA | | F | 25 | GG | NA | N | N |
| 300 | 47 | 76 | 44 | 129 | 89 | | F | 40 | GA | CC | N | N |
| 301 | 129 | 66 | 35 | 127 | 100 | | M | 57 | GA | CC | N | Y |
| 302 | 95 | 151 | 51 | 227 | 120 | | F | 76 | GA | CC | N | N |
| 303 | 92 | 106 | 77 | 201 | 108 | | F | 45 | AA | CC | N | N |
| 304 | 117 | 105 | 49 | 177 | 83 | | M | 43 | AA | CC | N | N |
| 305 | 151 | 92 | 56 | 178 | 87 | | F | 46 | GA | CC | N | N |
| 306 | 174 | 124 | 43 | 202 | 84 | | M | 36 | AA | CC | N | N |
| 307 | 164 | 108 | 51 | 192 | NA | | M | 70 | GA | CC | N | N |
| 308 | 207 | 109 | 38 | 188 | 104 | | F | 66 | GA | CC | N | Y |
| 309 | 189 | 81 | 53 | 172 | 111 | | F | 83 | GG | CC | N | N |
| 310 | 116 | 75 | 52 | 155 | 96 | | M | 62 | GG | CC | N | N |
| 311 | 100 | 127 | 63 | 210 | NA | | F | 53 | NA | CC | N | N |
| 312 | 98 | 165 | 61 | 246 | 91 | | F | 59 | AA | CC | N | Y |
| 313 | 139 | 104 | 23 | 155 | NA | | M | 44 | GA | CC | N | Y |
| 314 | 150 | 162 | 88 | 280 | 103 | | F | 72 | GA | CT | N | Y |
| 315 | 204 | 184 | 35 | 260 | 126 | | F | 53 | GG | CC | N | Y |
| 316 | 151 | 131 | 46 | 202 | 98 | | F | 30 | GA | CC | N | N |
| 317 | 528 | 169 | 32 | 261 | NA | | F | 80 | GG | CC | N | Y |
| 318 | 170 | 157 | 42 | 233 | 88 | | M | 70 | GA | CC | N | N |
| 319 | 73 | 155 | 67 | 237 | 106 | | F | 53 | NA | CC | N | N |
| 320 | 109 | 114 | 54 | 190 | 108 | | F | 34 | GA | CC | N | N |
| 321 | 150 | 133 | 47 | 210 | 114 | | F | 69 | AA | CC | N | N |
| 322 | 63 | 123 | 79 | 214 | 91 | | F | 28 | GA | CC | N | N |
| 323 | 145 | 176 | 36 | 242 | 129 | | M | 46 | GG | CC | N | Y |
| 324 | 88 | 143 | 41 | 202 | 96 | | F | 33 | NA | CC | N | N |
| 325 | 91 | 143 | 58 | 219 | 115 | | F | 47 | AA | CC | N | N |
| 326 | 130 | 110 | 83 | 219 | 116 | | F | 68 | GA | NA | N | N |
| 327 | 90 | NA | 41 | 93 | NA | | M | 28 | GA | NA | N | Y |
| 328 | 52 | 100 | 69 | 179 | 118 | | M | 48 | GG | CT | N | N |
| 329 | 159 | 174 | 44 | 237 | NA | | M | 34 | GA | NA | N | Y |
| 330 | 92 | 94 | 33 | 145 | 88 | | M | 76 | GA | CC | N | Y |
| 331 | 125 | 199 | 73 | 284 | NA | | F | 40 | AA | CC | N | Y |
| 332 | 174 | 95 | 46 | 176 | 102 | | F | 67 | AA | CC | N | N |
| 333 | 373 | 136 | 40 | 251 | 96 | | F | 70 | AA | CC | N | Y |
| 334 | 103 | 114 | 57 | 196 | 96 | | M | 55 | NA | CC | N | N |
| 335 | 195 | 125 | 51 | 215 | 104 | | M | 40 | GA | CC | N | N |
| 336 | 163 | 82 | 48 | 156 | NA | | M | 37 | GA | CC | N | N |
| 337 | 79 | 124 | 112 | 252 | 89 | | F | 68 | AA | CC | N | Y |
| 338 | 104 | 118 | 50 | 189 | 106 | | F | 64 | GG | CC | N | N |
| 339 | 162 | 73 | 63 | 161 | 111 | | M | 68 | GG | CC | N | N |
| 340 | 154 | 153 | 78 | 262 | NA | | F | 60 | GA | CT | N | Y |
| 341 | 288 | 74 | 23 | 151 | 114 | | M | 54 | GG | CC | N | Y |
| 342 | 112 | 135 | 34 | 191 | 97 | | M | 47 | GG | CC | N | Y |
| 343 | 129 | 146 | 99 | 281 | 118 | | F | 76 | NA | CC | N | Y |
| 344 | 331 | 106 | 42 | 194 | NA | | M | 37 | GA | CC | N | Y |
| 345 | 205 | 118 | 57 | 205 | 96 | | M | 60 | GA | CC | N | Y |
| 346 | 102 | 95 | 49 | 164 | 93 | | M | 37 | GA | CC | N | N |
| 347 | 131 | 126 | 53 | 205 | 125 | | F | 79 | NA | CC | N | N |
| 348 | NA | NA | NA | NA | NA | | M | 36 | GA | CC | N | Y |
| 349 | 157 | 98 | 37 | 166 | 110 | | M | 60 | NA | CC | N | Y |
| 350 | NA | NA | NA | NA | NA | | F | 40 | GA | CC | N | Y |
| TG, triglycerides;TC, total cholesterol; FBS, fasting blood sugar;M, male; F, female;SNP-1,rs1761667 polymorphism;SNP-2,rs1527483 polymorphism; T2DM, type 2 diabetes mellitus status; DYS, dyslipidemia status; Y, yes; N, no. | | | | | | | | | | | | |
