# Supplementary material for: Investigating the association of CD36 gene polymorphisms (rs1761667 and rs1527483) with T2DM and dyslipidemia: Statistical analysis, machine learning based prediction, and meta-analysis
Source: PLoS One. 2021 Oct 14;16(10):e0257857. doi: 10.1371/journal.pone.0257857 (PMC8516279; doi:10.1371/journal.pone.0257857)
Supplement: S2 Table — (DOCX) [file pone.0257857.s002.docx]

| **S2 Table.** Genotypic and allelic frequencies, and the exact tests for Hardy-Weinberg equilibrium (*n* = 309). | | | | | | |
| --- | --- | --- | --- | --- | --- | --- |
| **Polymorphism** | **N11** | **N12** | **N22** | **N1** | **N2** | ***p*-value** |
| rs1761667 | 85 | 164 | 60 | 334 | 284 | 0.25 |
| rs1527483 | 319 | 26 | 1 | 664 | 28 | 0.43 |
| N11, homozygous wild genotype; N12, heterozygous genotype; N22, homozygous mutant genotype; N1, wild allele; N2, mutant allele. | | | | | | |
