# Supplementary material for: Investigating the association of CD36 gene polymorphisms (rs1761667 and rs1527483) with T2DM and dyslipidemia: Statistical analysis, machine learning based prediction, and meta-analysis
Source: PLoS One. 2021 Oct 14;16(10):e0257857. doi: 10.1371/journal.pone.0257857 (PMC8516279; doi:10.1371/journal.pone.0257857)
Supplement: S3 Table — (DOCX) [file pone.0257857.s003.docx]

| **S3 Table.** Polymorphism rs1761667 and gender cross-classification interaction table. | | | | | | | |
| --- | --- | --- | --- | --- | --- | --- | --- |
|  | **Female** | | |  | **Male** | | |
| **Genotype** | **Control** | **T2DM** | **OR (95% CI)** |  | **Control** | **T2DM** | **OR (95% CI)** |
| GG | 9 | 22 | 1.00 |  | 19 | 14 | 0.56 (0.08-3.68) |
| GA | 25 | 43 | 1.64 (0.32-8.48) |  | 29 | 26 | 0.72 (0.13-3.98) |
| AA | 11 | 11 | 1.04 (0.13-8.37) |  | 10 | 14 | 2.78 (0.36-21.41) |
