# Supplementary material for: Investigating the association of CD36 gene polymorphisms (rs1761667 and rs1527483) with T2DM and dyslipidemia: Statistical analysis, machine learning based prediction, and meta-analysis
Source: PLoS One. 2021 Oct 14;16(10):e0257857. doi: 10.1371/journal.pone.0257857 (PMC8516279; doi:10.1371/journal.pone.0257857)
Supplement: S4 Table — (DOCX) [file pone.0257857.s004.docx]

| **S4 Table.** Polymorphism rs1527483 and gender cross-classification interaction table. | | | | | | | |
| --- | --- | --- | --- | --- | --- | --- | --- |
| **Genotype** | **Female** | | |  | **Male** | | |
|  | **Control** | **T2DM** | **OR (95% CI)** |  | **Control** | **T2DM** | **OR (95% CI)** |
| CC | 51 | 73 | 1.00 |  | 63 | 50 | 0.55 (0.22-1.40) |
| CT | 3 | 6 | 1.08 (0.06-19.01) |  | 5 | 5 | 1.92 (0.28-13.16) |
| TT | 0 | 0 | --- |  | 1 | 0 | 0.00 |
