# Supplementary material for: Investigating the association of CD36 gene polymorphisms (rs1761667 and rs1527483) with T2DM and dyslipidemia: Statistical analysis, machine learning based prediction, and meta-analysis
Source: PLoS One. 2021 Oct 14;16(10):e0257857. doi: 10.1371/journal.pone.0257857 (PMC8516279; doi:10.1371/journal.pone.0257857)
Supplement: S5 Table — (DOCX) [file pone.0257857.s005.docx]

| **S5 Table.** Haplotype frequencies estimation (*n* = 350). | | |
| --- | --- | --- |
| **Polymorphism** | | **Total frequency** |
| **rs1761667** | **rs1527483** |  |
| G | C | 0.5094 |
| A | C | 0.4501 |
| G | T | 0.0333 |
| A | T | 0.0072 |
