# Supplementary material for: Investigating the association of CD36 gene polymorphisms (rs1761667 and rs1527483) with T2DM and dyslipidemia: Statistical analysis, machine learning based prediction, and meta-analysis
Source: PLoS One. 2021 Oct 14;16(10):e0257857. doi: 10.1371/journal.pone.0257857 (PMC8516279; doi:10.1371/journal.pone.0257857)
Supplement: S6 Table — (DOCX) [file pone.0257857.s006.docx]

| **S6 Table.** Haplotype and gender cross-classification interaction. | | | |
| --- | --- | --- | --- |
| **Haplotype** | **Frequency** | **OR (95% CI)** | |
|  |  | **Female** | **Male** |
| GC | 0.5069 | 1.00 | 0.24 (0.04 - 1.30) |
| AC | 0.4523 | 1.15 (0.42 - 3.16) | 0.64 (0.17 - 2.46) |
| GT | 0.0367 | 1.13 (0.05 - 25.66) | 0.88 (0.12 - 6.46) |
| rare | 0.0041 | 1.30 (0.00 - 649.78) | --- |
| Interaction *p*-value: 0.64 | | | |
