# Supplementary material for: Investigating the association of CD36 gene polymorphisms (rs1761667 and rs1527483) with T2DM and dyslipidemia: Statistical analysis, machine learning based prediction, and meta-analysis
Source: PLoS One. 2021 Oct 14;16(10):e0257857. doi: 10.1371/journal.pone.0257857 (PMC8516279; doi:10.1371/journal.pone.0257857)
Supplement: S7 Table — (DOCX) [file pone.0257857.s007.docx]

| **S7 Table.** Polymorphism rs1761667 and gender cross-classification interaction table. | | | | | | | |
| --- | --- | --- | --- | --- | --- | --- | --- |
|  | **Female** | | |  | **Male** | | |
| **Genotype** | **No-dyslipidemia** | **Dyslipidemia** | **OR (95% CI)** |  | **No-dyslipidemia** | **Dyslipidemia** | **OR (95% CI)** |
| GG | 12 | 19 | 1.00 |  | 11 | 22 | 5.28 (1.08-25.88) |
| GA | 32 | 36 | 0.79 (0.21-2.96) |  | 20 | 35 | 1.77 (0.45-7.04) |
| AA | 10 | 12 | 1.91 (0.37-9.78) |  | 10 | 14 | 2.05 (0.42-9.98) |
| Interaction *p*-value: 0.38 | | | | | | | |
