# Supplementary material for: Investigating the association of CD36 gene polymorphisms (rs1761667 and rs1527483) with T2DM and dyslipidemia: Statistical analysis, machine learning based prediction, and meta-analysis
Source: PLoS One. 2021 Oct 14;16(10):e0257857. doi: 10.1371/journal.pone.0257857 (PMC8516279; doi:10.1371/journal.pone.0257857)
Supplement: S8 Table — (DOCX) [file pone.0257857.s008.docx]

| **S8 Table.** Polymorphism rs1527483 and gender cross-classification interaction. | | | | | | | |
| --- | --- | --- | --- | --- | --- | --- | --- |
| **Genotype** | **Female** | | |  | **Male** | | |
|  | **No-dyslipidemia** | **dyslipidemia** | **OR (95% CI)** |  | **No-dyslipidemia** | **dyslipidemia** | **OR (95% CI)** |
| CC | 59 | 65 | 1.00 |  | 43 | 70 | 2.48 (1.15-5.36) |
| CT | 5 | 4 | 1.26 (0.20-7.92) |  | 4 | 6 | 3.60 (0.48-26.73) |
| TT | 0 | 0 | --- |  | 1 | 0 | 0.00 |
| Interaction *p*-value: 0.92 | | | | | | | |
