# Supplementary material for: Investigating the association of CD36 gene polymorphisms (rs1761667 and rs1527483) with T2DM and dyslipidemia: Statistical analysis, machine learning based prediction, and meta-analysis
Source: PLoS One. 2021 Oct 14;16(10):e0257857. doi: 10.1371/journal.pone.0257857 (PMC8516279; doi:10.1371/journal.pone.0257857)
Supplement: S9 Table — (DOCX) [file pone.0257857.s009.docx]

| **S9 Table.** Frequencies and numbers (in brackets) of alleles and genotypes of both polymorphisms for all studies involved in meta-analysis. | | | | | | |
| --- | --- | --- | --- | --- | --- | --- |
| **Polymorphism** | **Study** | **Controls frequency wild allele** | | **Controls**  **frequency mutant allele** | | **Total number** |
| rs1761667 | Banerjee *et al.* | G = 0.64 (193) | | A= 0.36 (107) | | 300 |
|  | The present study | G = 0.53 (110) | | A= 0.47 (96) | | 206 |
| rs1527483 | Banerjee *et al.* | C = 0.89 (268) | | T = 0.11 (32) | | 300 |
|  | The present study | C = 0.96 (236) | | T= 0.04 (10) | | 246 |
|  |  | **Patients frequency wild allele** | | **Patients frequency mutated allele** | |  |
| rs1761667 | Banerjee *et al.* | G = 0.60 (301) | | A= 0.40 (199) | | 500 |
|  | The present study | G = 0.54 (141) | | A= 0.46 (119) | | 260 |
| rs1527483 | Banerjee *et al.* | C = 0.88 (439) | | T = 0.12 (61) | | 500 |
|  | The present study | C = 0.96 (257) | | T= 0.04 (11) | | 268 |
|  |  | **Controls frequency wild homozygous** | **Controls frequency heterozygous** | | **Controls frequency mutant homozygous** |  |
| rs1761667 | Banerjee *et al.* | GG = 0.40 (60) | GA = 0.49 (73) | | AA = 0.11 (17) | 150 |
|  | The present study | GG = 0.27 (28) | GA = 0.52 (54) | | AA = 0.20 (21) | 103 |
| rs1527483 | Banerjee *et al.* | CC = 0.80 (120) | CT = 0.19 (28) | | TT = 0.01 (2) | 150 |
|  | The present study | CC = 0.93 (114) | CT = 0.65 (8) | | TT = 0.01 (1) | 123 |
|  |  | **Patients frequency wild homozygous** | **Patients frequency heterozygous** | | **Patients frequency mutant homozygous** |  |
| rs1761667 | Banerjee *et al.* | GG = 0.22 (56) | GA = 0.76 (189) | | AA = 0.02 (5) | 250 |
|  | The present study | GG = 0.28 (36) | GA = 0.53 (69) | | AA = 0.19 (25) | 130 |
| rs1527483 | Banerjee *et al.* | CC = 0.76 (190) | CT = 0.24 (59) | | TT = 0.00 (1) | 250 |
|  | The present study | CC = 0.92 (123) | CT = 0.08 (11) | | TT = 0.00 (0) | 134 |
